# Systematic analysis of the role of SLC52A2 in multiple human cancers

**DOI:** 10.1186/s12935-021-02432-7

**Published:** 2022-01-06

**Authors:** Lilong Zhang, Man Li, Zhoujun Cui, Dongqi Chai, Yongjun Guan, Chen Chen, Weixing Wang

**Affiliations:** 1 Department of General Surgery, Renmin Hospital of Wuhan University, No. 238, Jiefang Road, Wuchang District, Wuhan, 430060 Hubei Province China; 2 Central Laboratory, Renmin Hospital of Wuhan University, No. 238, Jiefang Road, Wuchang District, Wuhan, 430060 Hubei Province China; 3 Department of General Surgery, People’s Hospital of Rizhao, 126 Tai’an Road, Donggang District, Rizhao, 276800 Shandong Province China

**Keywords:** SLC52A2, Pan-cancer, Hepatocellular, Carcinoma, Bioinformatics, Prognosis

## Abstract

**Background:**

In humans, riboflavin must be obtained through intestinal absorption because it cannot be synthesized by the body. SLC52A2 encodes a membrane protein belonging to the riboflavin transporter protein family and is associated with a variety of diseases. Here, we systematically explore its relevance to multiple human tumors.

**Methods:**

We analyzed the association of SLC52A2 with 33 tumors using publicly available databases such as TCGA and GEO. We verified the SLC52A2 expression in hepatocellular carcinoma, gastric cancer, colon cancer, and rectal cancer using immunohistochemistry.

**Results:**

We report that SLC52A2 was highly expressed in almost all tumors, and the immunohistochemical results in the hepatocellular, gastric, colon, and rectal cancers were consistent with the above. SLC52A2 expression was linked to patient overall survival, disease-specific survival, progression-free interval, diagnosis, mutations, tumor mutational burden, microsatellite instability, common immune checkpoint genes, and immune cells infiltration. Enrichment analysis showed that SLC52A2 was mainly enriched in oocyte meiosis, eukaryotic ribosome biogenesis, and cell cycle. In hepatocellular carcinoma, the SLC52A2 expression is an independent prognostic factor. The SNHG3 and THUMPD3-AS1/hsa-miR-139-5p-SLC52A2 axis were identified as potential regulatory pathways in hepatocellular carcinoma.

**Conclusion:**

In conclusion, we have systematically described for the first time that SLC52A2 is closely associated with a variety of tumors, especially hepatocellular carcinoma.

**Supplementary Information:**

The online version contains supplementary material available at 10.1186/s12935-021-02432-7.

## Introduction

Riboflavin (also called vitamin B2) is a water-soluble vitamin found in various foods. Flavin adenine dinucleotide and flavin mononucleotide are their most important biologically active forms, which are key to carbohydrates, amino acids, and lipid metabolism [1]. And riboflavin deficiency causes growth impairment [2]. Bacteria, fungi, and plants can synthesize riboflavin from GTP and ribulose 5Pi. On the contrary, higher organisms lose the ability to synthesize this vitamin and must be obtained by intestinal absorption [3]. Riboflavin is absorbed mainly in the small intestine. Milk, dairy products, meat, fish, and dark-green vegetables are essential sources of riboflavin [4].

It was found that the absorption of riboflavin and regulation of riboflavin tissue distribution was mainly dependent on specific transporters of the human solute carrier family 52 (SLC52) [5]. Currently, this family has been confirmed to include SLC52A1, SLC52A2, SLC52A3 [5]. SLC52A2 (also known as GPR172A, RFT3, RFVT2, PAR1) gene, located at locus 8q24.3, encodes a protein of 445-amino acid [6–8]. SLC52A2-mediated riboflavin uptake has been confirmed to be Na^+^-independent, Cl^−^-independent, and pH-independent, which has been indicated that SLC52A2 is crucial for tissue distribution of riboflavin [7, 9].

It has been found that the organism is susceptible to riboflavin deficiency under pathological stress. Such riboflavin deficiency has been considered a risk factor for cancer, cardiovascular disease, and neurodegeneration [1]. Cheng et al. firstly reported the study of cancer-related copy number amplification and overexpression of SLC52A2. They found that, compared with matched adjacent noncancerous, SLC52A2 showed increased copy number in 44% gastric cancer tissues and its expression was upregulated in 56% gastric cancer tissues [10].

In this study, bioinformatic analysis was conducted to explore the SLC52A2 expression in various human tumors and its possible association with cancer, which was confirmed by immunohistochemistry, to provide more information to better understand the importance of SLC52A2 in various cancers.

## Materials and methods

### Data collection and processing

Xena Browser was used to obtain the Genotype-Tissue Expression (GTEx) data and The Cancer Genome Atlas (TCGA) data. The Cancer Cell Line Encyclopedia (CCLE) database was used to acquire the SLC52A2 expression in cancer cell lines. The ONCOMINE database was selected to further understand the SLC52A2 expression in multiple cancers, with “p-value = 0.05” and “fold change = 1.5”. The TISIDB database was used to examine SLC52A2 expression in different immune subtypes in tumor samples [11]. The cBioPortal database and Catalogue of Somatic Mutations in Cancer (COSMIC) database were used to estimate the mutation levels of SLC52A2 in different human cancers [12, 13]. GSE121248, GSE112790, GSE39791, GSE55092, GSE62232, and GSE102079 datasets were downloaded via GEO. Tumor IMmune Estimation Resource (TIMER) was used to estimate the association between SLC52A2 and immune cell infiltration [14].

### mRNA expression of SLC52A2 in multiple human cancers

The “gganatogram” and “ggpubr” R software packages were used to study the SLC52A2 expression in 31 normal human tissues. And, unpaired t-test was conducted to estimate the differences between males and females in normal tissues. For all TCGA data, the number of normal samples per tumor was counted, and tumors with the number of normal samples ≥ 5 were selected. Then, the ‘ggpubr’ R software package was used to estimate the expression of differences by Wilcoxon’s test between normal and tumor tissues. Besides, we also investigated the association between clinical stages, grade, gender, race, tumor status, and SLC52A2 expression in 33 cancers by “limma” and “ggpubr” R software packages.

### Cox regression analysis, survival analysis, and diagnosis analysis

The association between SLC52A2 expression and overall survival (OS), disease-specific survival (DSS), progression-free interval (PFI) was assessed using phenotype and survival data of TCGA data. The difference in survival between the high and low expression groups was estimated by the Kaplan-Meyer method using the “survival” and “survivor” R packages. The Cox proportional hazard regression analysis was used to evaluate the hazard ratio by “survival”, “survminer”, “limmu” and “forestplot” R software package. Also, the AUC value was calculated by using the “pROC” R software package.

### Tumor mutational burden and microsatellite instability

To calculate the number of mutations in the 33 cancer samples, the tumor mutational burden (TMB) was estimated and divided by the total length of the exons. The microsatellite instability (MSI) score was acquired from TCGA. We used Spearman’s method to estimate the correlation analysis between the SLC52A2 expression and TMB or MSI. Both indicators were displayed on a radar map by the “fmsb” R software package.

### Immune cell infiltration enrichment and co-expression analysis of some common immune checkpoint genes

Co-expression analysis of immune marker set and some common immune checkpoint genes was conducted with “limma” R software packages and visualized using “reshape2” and “RColorBrewer” R software packages. On the TIMER2.0 platform [15], we used CIBERSORT and TIDE algorithms to analyze the relationship between SLC52A2 expression and immune cell infiltration.

### SLC52A2-related gene enrichment analysis

The “Similar Gene Detection” module of GEPIA2 was used to acquire the SLC52A2-related genes (R > 0.4). Then, the protein interaction network of the above genes was established using the STRING website [16]. Both Kyoto Encyclopedia of Genes and Genomes (KEGG) and Gene Ontology (GO) enrichment pathway as well as functional annotation analysis of SLC52A2 in 33 types of tumors were conducted using “clusterProfiler”, “org.Hs.eg.db”, “enrichplot”, “DOSE”, “colorspace”, “stringi” and “ggplot2” R software package. In addition, according to the expression level of SLC52A2, patients in the LIHC-TCGA cohort were divided into high and low expression groups, and then KEGG (c2.cp.kegg.v7.4.symbols.gmt) and GO (c5.go.bp.v7.4.symbols.gmt, c5.go.cc.v7.4.symbols.gm-t, c5.go.mf.v7.4.symbols.gmt) enrichment analysis was performed using GSEA_4.1.0 software.

### Prognostic analysis of SLC52A2 in LIHC

We conducted univariate and multivariate Cox regression analyses to determine the appropriate terms for constructing the nomogram by TCGA database. Forests were used to display the P-value, HR, and 95% confidence interval for each variable by “forestplot” R software package. According to the results of a multivariate Cox proportional hazards analysis, a nomogram was developed to predict the 1-, 2-, 3- and 5-year OS.

### Prediction of upstream lncRNA and miRNAs of SLC52A2 in LIHC

starBase is a database for exploring miRNA-related studies [17]. Upstream binding miRNAs of SLC52A2 were predicted using the miRNA-Target module (miRNA-mRNA option), and the lncRNAs upstream of hsa-miR-139-5p were explored using the miRNA-Target module (miRNA-lncRNA option) in starbase. Pan-cancer module in starbase was used to analyze the miRNA differential expression, miRNA survival analysis, and miRNA-target coexpression. The GEPIA2 tool was used to estimate the lncRNA differential expression and lncRNA survival analysis.

### Immunohistochemical staining

We collected tumor and paraneoplastic tissues from LIHC (40 cases), STAD (20 cases), COAD (20 cases), and READ (20 cases) from January 2018 to December 2020 at the Renmin Hospital of Wuhan University, China. Immunohistochemistry analysis was performed on these cancers. Briefly, the paraffin sections were placed on a stainless shelf and incubated at 70 °C for 80 min in an oven. After xylene deparaffinization twice (20 min each), gradient ethanol hydration (100%, 95%, 85%, 75%, 5%, 5 min each), and deionized water washing. The sections were put on boiling of sodium citrate buffer (pH 9.0) and further boiled for 4 min in a pressure cooker for antigen retrieval. After cooling, the slides were blocked with 3% hydrogen peroxide solution for 15 min. The sections were incubated overnight at 4 °C with anti-SLC52A2 antibody (rabbit, Solarbio, 1:100) in a humidity box, then incubated with a second antibody. The staining results were visualized using the 3,5-diaminobenzidine. Immunostained sections were characterized quantitatively as the integrated optical density (IOD) by digital image analysis using the ImageJ 1.53e software (Wayne Rasband and contributors National Institutes of Health, USA). Images were obtained with an fluorescence microscope (Olympus BX63, Tokyo, Japan). A series of 5 random images on each section was taken for each immunostained parameter to obtain a mean value for statistical comparison. The cutoff for the definition of high expression group or low expression group was the median value. Data were expressed as the mean ± standard deviation. Statistical measurements were performed using the SPSS 21.0 statistical software (SPSS Inc., Chicago, USA).

### Quantitative reverse transcription PCR (qRT-PCR)

Among the above LIHC patients, 15 of them had their tumor tissues and paraneoplastic tissues frozen at − 80 °C. Therefore, we used this part of patients for the detection of the SLC52A2 mRNA level. Total RNA was extracted using Trizol RNA extraction kit (Servicebio) according to the manufacture’s instructions. RNA was reverse-transcribed to complementary DNA according to the manufacturer’s instructions of Revert Aid First Strand cDNA Synthesis Kit (Thermo Fisher Scientific). qRT-PCR was performed with the primers for SLC52A2 (F: AGGTGGGAAAAGAACTGGC; R: CAAGCACAGAGACGTAAGAGG) (Tsingke Biotechnology Co., Ltd.) and β-actin (F: CCTTCCTGGGCATGGAGTC; R: TGATCTTCATTGTGCTGGGTG) (Tsingke Biotechnology Co., Ltd.) using 2 × SYBR Green qPCR Master Mix (None ROX) (Servicebio). The reaction conditions were pre-denaturation at 95 °C for 30 s, followed by 40 cycles of denaturation at 95 ℃ for 15 s and annealing/extension at 60 ℃ for 30 s. The relative gene expression level was computed by the 2^−ΔΔCT^ way. β-actin was used as an internal reference for normalization.

See Additional file 1: Table S1 for details of the database sites used in this study. Table 1 provided the full cancer type name corresponding to each abbreviation listed in the legend and the text.

**Table 1:** Full names and abbreviations of the 33 cancers in the TCGA database

Abbreviation	Full name
ACC	Adrenocortical carcinoma
BLCA	Bladder urothelial carcinoma
BRCA	Breast invasive carcinoma
CESC	Cervical squamous cell carcinoma and endocervical adenocarcinoma
CHOL	Cholangiocarcinoma
COAD	Colon adenocarcinoma
DLBC	Lymphoid neoplasm diffuse large B-cell lymphoma
ESCA	Esophageal carcinoma
GBM	Glioblastoma multiforme
HNSC	Head and neck squamous cell carcinoma
KICH	Kidney chromophobe
KIRC	Kidney renal clear cell carcinoma
KIRP	Kidney renal papillary cell carcinoma
LAML	Acute myeloid leukemia
LGG	Brain lower grade glioma
LIHC	Liver hepatocellular carcinoma
LUAD	Lung adenocarcinoma
LUSC	Lung squamous cell carcinoma
MESO	Mesothelioma
OV	Ovarian serous cystadenocarcinoma
PAAD	Pancreatic adenocarcinoma
PCPG	Pheochromocytoma and paraganglioma
PRAD	Prostate adenocarcinoma
READ	Rectum adenocarcinoma
SARC	Sarcoma
SKCM	Skin cutaneous melanoma
STAD	Stomach adenocarcinoma
TGCT	Testicular germ cell tumors
THCA	Thyroid carcinoma
THYM	Thymoma
UCEC	Uterine corpus endometrial carcinoma
UCS	Uterine carcinosarcoma
UVM	Uveal melanoma

## Results

### mRNA expression of SLC52A2 in multiple human cancers

**Fig. 1:**
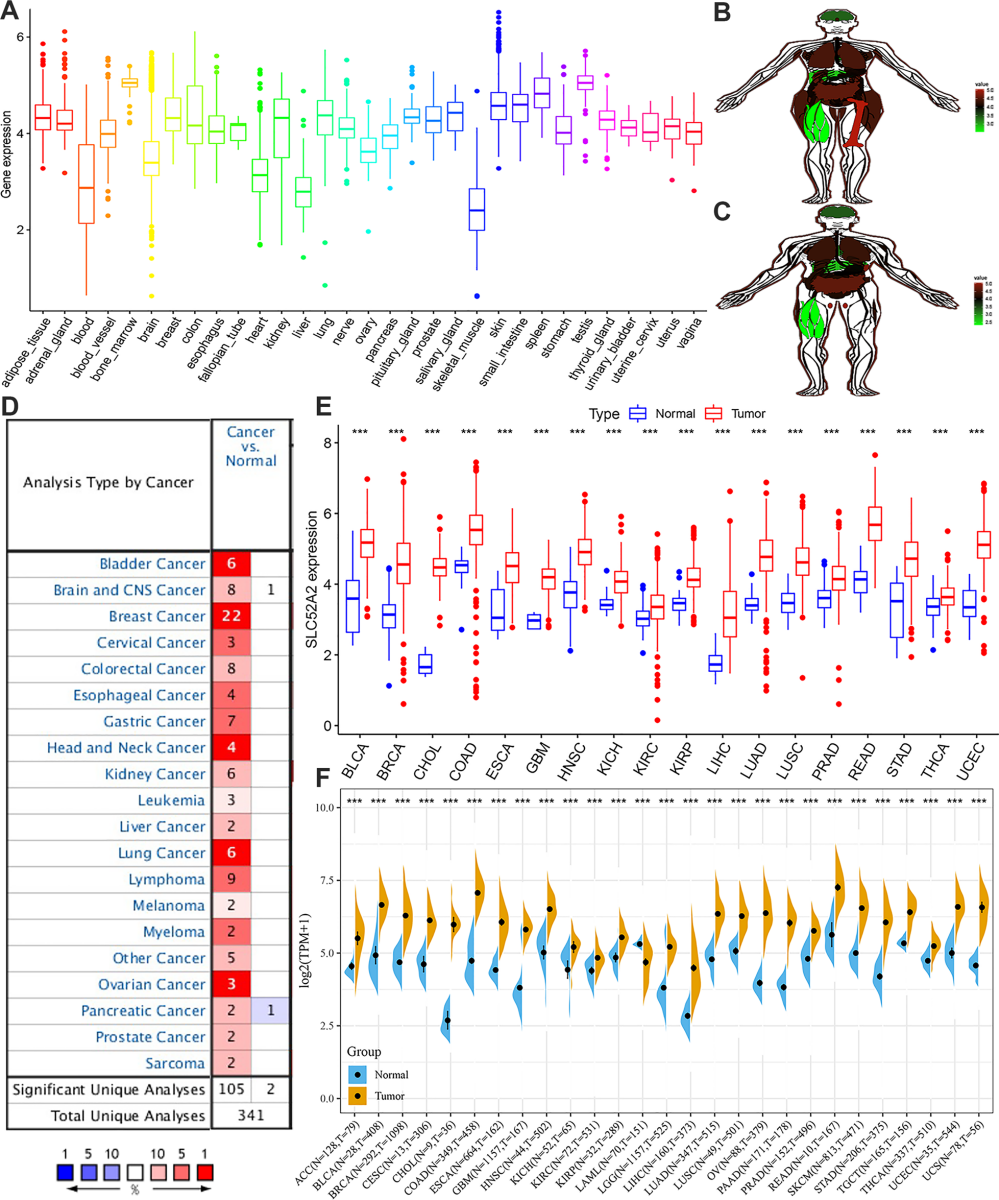
mRNA expression of SLC52A2 gene in various cancers and its corresponding normal tissues. **A** Normal tissues expression. **B** Female expression anatomy diagram. **C** Male expression anatomy diagram. **D** Oncomine dataset. **E** TCGA dataset. **F** TCGA and GTEx dataset (***P < 0.001)

First, we used the GTEx database to analyze the SLC52A2 expression in 31 types of normal tissues. The results revealed that the SLC52A2 expression was the highest in the bone marrow, spleen and testis tissues, while the lowest in the skeletal muscle, blood, and liver (Fig. 1A). Besides, the expression of SLC52A2 in adipose tissues and skeletal muscle tissues was significantly higher in females than in males (Fig. 1B, C; Additional file 4: Fig. S1A). Subsequently, the CCLE database was used to estimate the SLC52A2 expression in various cancer cell lines. The results demonstrated that the SLC52A2 was expressed in all 38 kinds of tumor cell lines. Specifically, the meningioma cell lines and colorectal cell lines express the highest levels of SLC52A2. The diffuse large B-cell lymphoma cell lines and T-cell acute lymphoblastic leukemia cell lines were the lowest (Additional file 4: Fig. S1B).

Next, we analyzed the difference in mRNA expression of SLC52A2 between various cancer and normal tissues using the ONCOMINE database. We found that the SLC52A2 expression was more elevated in all cancers (Fig. 1D). To further estimate SLC52A2 expression in multiple human cancers, we examined mRNA sequencing data from the TCGA database. After selecting tumors with the number of normal sample size ≥ 5, we confirmed the above findings with a significant upregulation of SLC52A2 expression in all cancers compared to normal tissues, including BLCA, BRCA, CHOL, COAD, ESCA, GBM, HNSC, KICH, KIRC, KIRP, LUAD, LIHC, LUSC, PRAD, READ, STAD, THCA, and UCEC (Fig. 1E). Because of the small number of normal tissues in the TCGA database, we further combined the GTEx database and the TCGA database by the pan-cancer platform SangerBox. In addition to confirming the above TCGA results, we also found that SLC52A2 was significantly highly expressed in ACC, CESC, LGG, OV, PAAD, SKCM, TCGT, and UCS, while significantly lowly expressed in LAML than normal tissues (Fig. 1F).

### Correlation analysis of SLC52A2 expression and the clinical features of multiple cancers

**Fig. 2:**
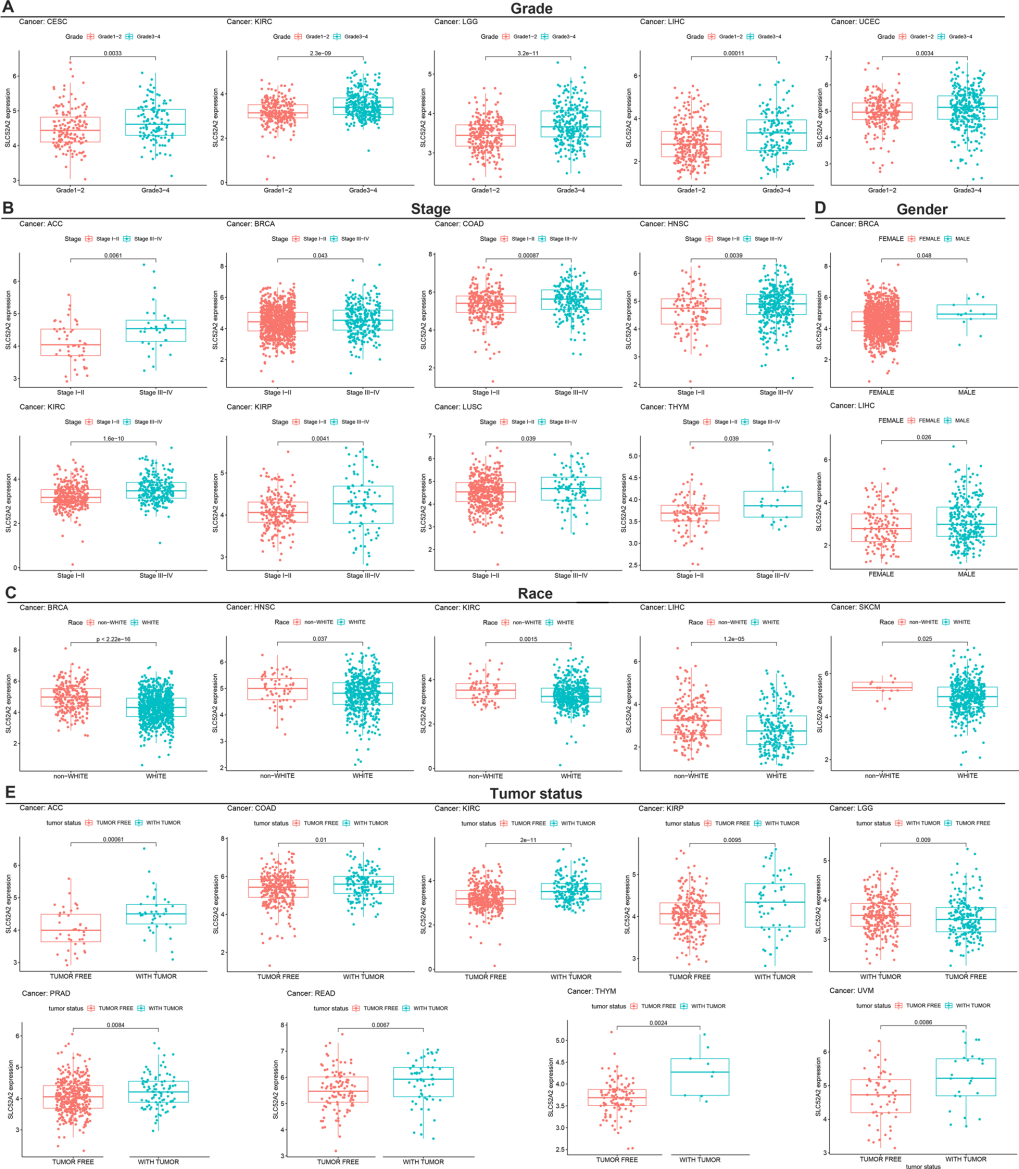
Correlation of SLC52A2 mRNA expression levels with clinical characteristics. Correlation between SLCC52A2 and grade (**A**), stage (**B**), race (**C**), gender (**D**), and tumor status (**E**)

We estimated the association between the SLC52A2 expression and the clinical features across human cancers using TCGA data. We found that SLC52A2 expression was significantly higher in grade 3–4 patients than in grade1-2 in CESC, KIRC, LGG, LIHC, UCEC (Fig. 2A). In ACC, BRCA, COAD, HNSC, KIRC, KIRP, LUSC, and THYM, we revealed that patients with stage III − IV expressed significantly higher than the patients with stage I − II (Fig. 2B). As for the race, we found that patients with the non-White population (Native Hawaiian or other Pacific Islander, Pacific Islander, Black or African American, American Indian or Alaska Native, and Asian) expressed significantly higher than the patients with the White population in BRCA, HNSC, KIRC, LIHC, and SKCM (Fig. 2C). We also found that the expression of SLC52A2 was significantly higher in males than in females in BRCA and LIHC (Fig. 2D). Finally, we analyzed the correlation between SLC52A2 expression and tumor status. This result shows that compared with tumor-free, SLC52A2 expression in the presence of tumors was higher in ACC, COAD, KIRC, KIRP, LGG, PRAD, READ, THYM, and UVM (Fig. 2E). Thorsson et al.[18]. found six stable and reproducible immune subtypes, including wound healing (C1), IFN-γ dominant (C2), inflammatory (C3), lymphocyte deplete (C4), immunologically quiet (C5), and TGF-β dominant (C6), which cover almost all cancers. These subtypes were related to prognosis, genetic, and immune-modulatory alterations that may shape the specific types of immune environments. Here, we examined the expression of SLC52A2 in different immune subtypes in multiple cancers and found a significant difference in BRCA, KIRC, KICH, TGCT, LIHC, LUAD, LUSC, OV, PAAD, PRAD, SARC, and STAD (Additional file 5: Fig. S2). Interestingly, SLC52A2 expression was lowest in the C3 immune subtype and higher in the C4 immune subtype.

### Prognostic analysis of SLC52A2 in multiple human cancers

**Fig. 3:**
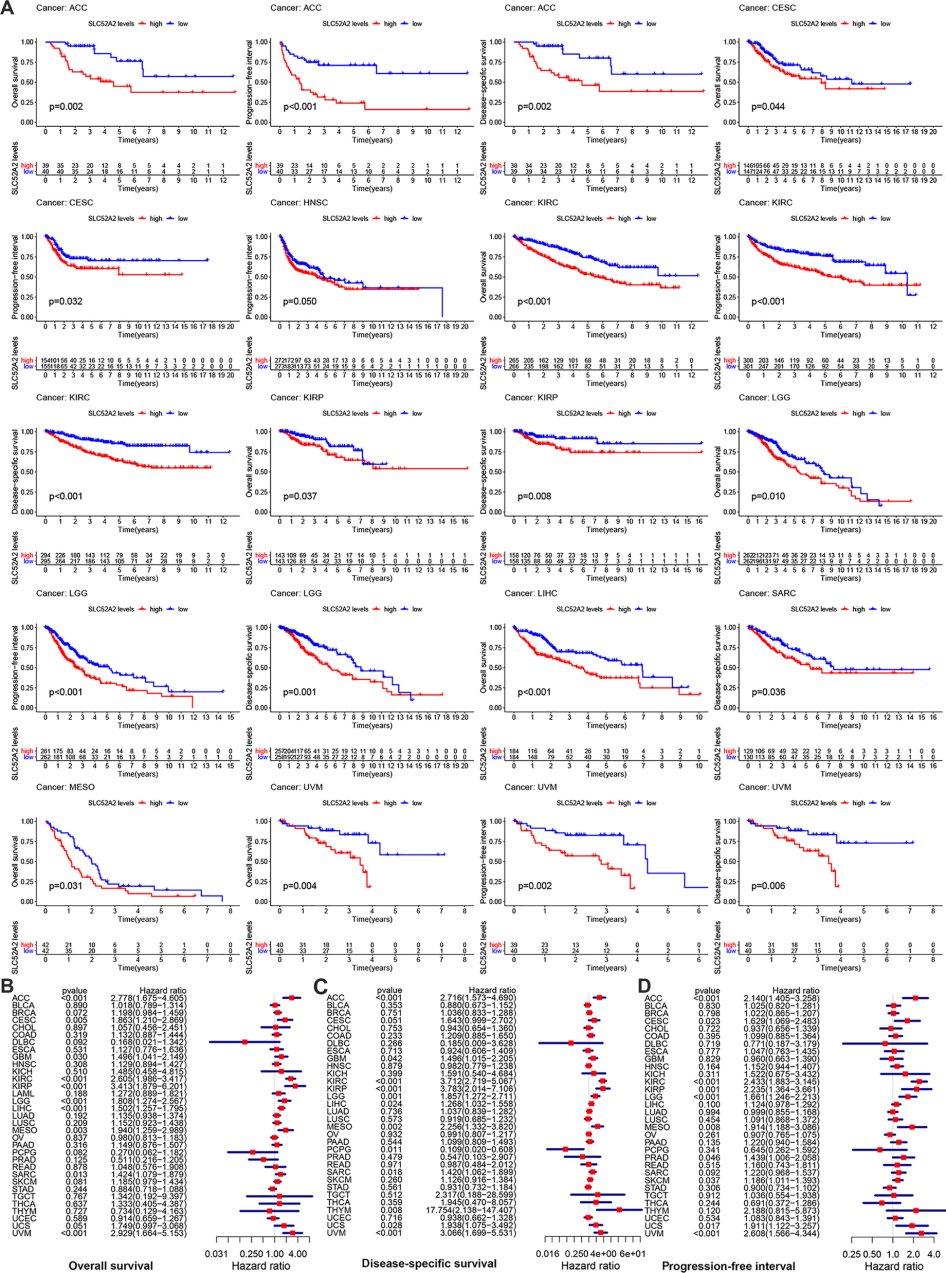
Relationship between SLC52A2 expression and overall survival (OS), disease-specific survival (DSS), progression-free interval (PFI) in 33 tumors. The survival curves were calculated using Kaplan–Meier survival methods (**A**) and the forest plots were calculated using univariate Cox regression (**B**–**D**)

TCGA database was used to estimate the correlation between SLC52A2 expression and survival in multiple human cancers. When the median SLC52A2 expression value of 33 types of human cancers was chosen as the cut-off value by Kaplan–Meier analysis, the results have shown that the high expression of SLC52A2 was related to poor survival in ACC (OS, DSS, PFI), CESC (OS, PFI), HNSC (PFI), KIRC (OS, DSS, PFI), KIRP (OS, DSS), LGG (OS, DSS, PFI), LIHC (OS), SARC (DSS), MESO (OS) and UVM (OS, DSS, PFI), whereas no statistical differences existed in other tumors (Fig. 3A).

We further used Cox analysis to explore the SLC52A2-related survival (OS, DSS, and PFI) (Fig. 3B–D). We thus identified that SLC52A2 acted as a detrimental prognostic factor in ACC (OS, DSS, PFI), CESC (OS, PFI), GBM (OS, DSS), KIRC (OS, DSS, PFI), KIRP (OS, DSS, PFI), LGG (OS, DSS, PFI), LIHC (OS, DSS), MESO (OS, DSS, PFI), PRAD (PFI), SARC (OS, DSS), SKCM (PFI), THYM (DSS), UCS (PFI) and UVM (OS, DSS, PF). While SLC52A2 expression was a protective prognostic factor in PCPG (DSS) (Fig. 3C).

Finally, we also explored the diagnostic value of SLC52A2 expression in pan-cancer. We selected cancers from the TCGA database with the number of normal controls in more than 40 samples and calculated the ROC curves and AUC. The AUC of the ROC had values higher than 0.70 in all analyses, including BRCA (AUC = 0.943), COAD (AUC = 0.913), HNSC (AUC = 0.941), KIRC (AUC = 0.706), LIHC (AUC = 0.954), LUAD (AUC = 0.954), LUSC (AUC = 0.966), PRAD (AUC = 0.794), and THCA (AUC = 0.721) (Additional file 6: Fig. S3), indicating a reasonable predictive model performance.

### SLC52A2 mutations in multiple human cancers

We analyzed the alteration frequency of SLC52A2 in pan-cancer by the cBioPortal database. The results indicated that SLC52A2 was altered in 691 of the 10,953 patients (6%) (Additional file 7: Fig. S4A). The highest alteration frequency was associated with amplification, followed by deep deletion and mutation. Among pan-cancer, OV had the highest alteration ratio with 26.2%. Additional file 7: Fig. S4B further shows the type, sites, and case numbers of mutations in the SLC52A2 gene. 72 mutation sites (including 58 missense, 9 truncating, 4 fusion, and 1 inframe) located between amino acids 0 and 445 were identified in SLC52A2. We further analyzed the mutation distribution of the SLC52A2 gene in multiple human cancers by the COSMIC database. The results showed that missense mutations and synergistic mutations were the main mutation types in cancers (Additional file 7: Fig. S4C). The C > T and G > A mutations were most common in the SLC52A2 coding chain, and A > G and G > T mutations were also common (Additional file 7: Fig. S4C). Besides, we estimated the correlation between genetic alteration of SLC52A2 and the prognosis of cancers by the cBioPortal database. The data of Additional file 7: Fig. S4D–G indicate that cancer patients with altered levels of SLC52A2 showed poor prognosis in the progress-free interval and disease-free interval compared with cases without SLC52A2 alteration.

### Association of SLC52A2 expression with MSI, TMB and some common immune checkpoint genes in multiple human cancers

**Fig. 4:**
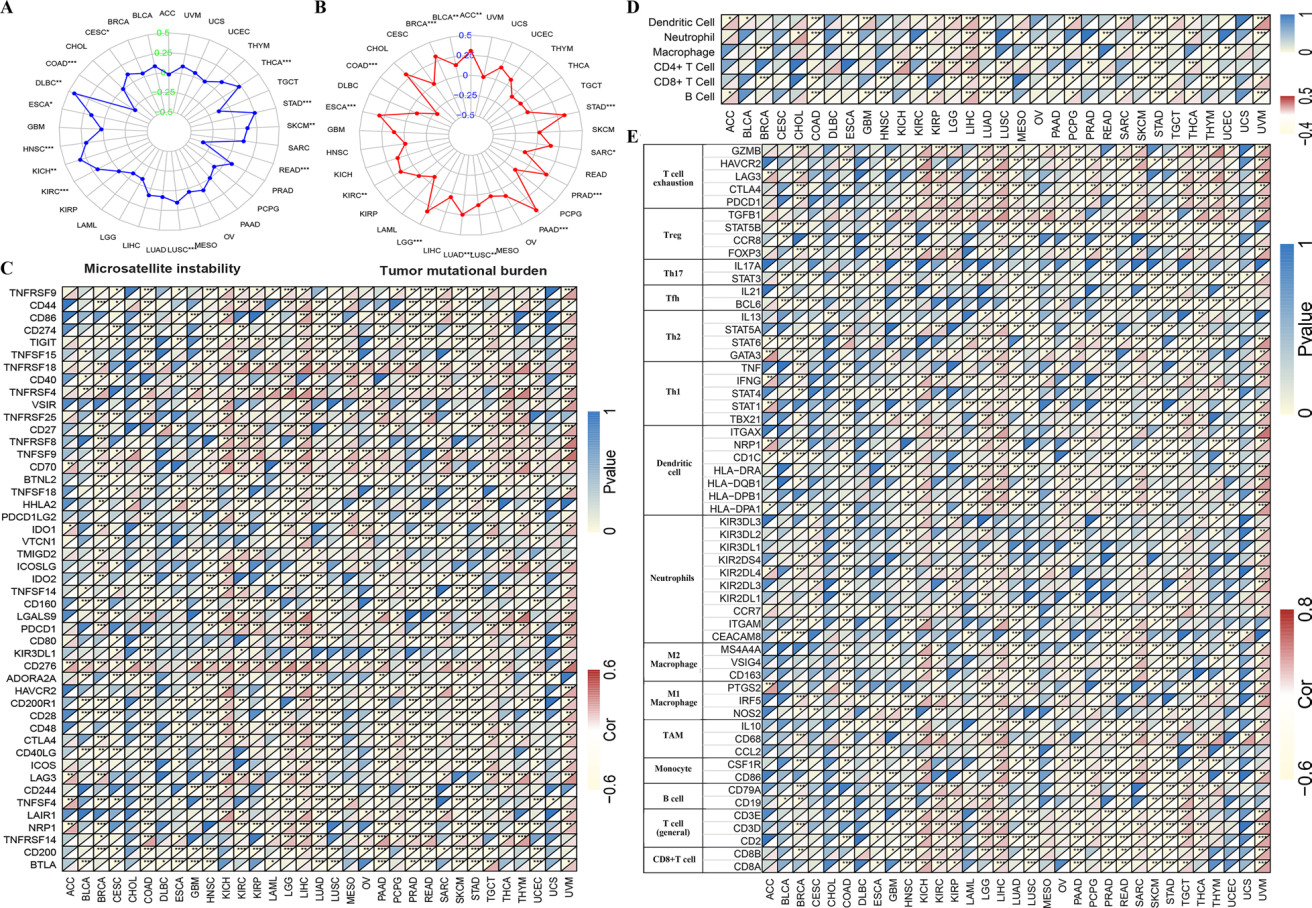
Relation between microsatellite instability (**A**), tumor mutational burden (**B**), some immune checkpoints (**C**), immune cell infiltration (**D**), immune marker sets (**E**), and SLC52A2 mRNA expression in various tumors in TCGA database. The lower triangle in each tile indicates coefficients calculated by Pearson’s correlation test, and the upper triangle indicates log_10_-transformed *P*-value (***P < 0.001, **P < 0.01, *P < 0.05)

Currently, MSI and TMB status are important indicators of prognosis for patients with tumors and can facilitate the choice of treatment, especially the application of immune checkpoint inhibitors (ICIs) [19]. Therefore, we estimated the relationship between the MSI, TMB, and SLC52A2 expression in pan-cancer. Our results revealed that SLC52A2 expression was positively associated with MSI in THCA, STAD, SKCM, LUSC, KIRC, KICH, HNSC, ESCA, DLBC, and CESC, but negatively associated with MSI in READ and COAD (Fig. 4A). As for TMB, our results also demonstrated that the SLC52A2 expression was significantly related to TMB in 13 human cancers. Among these, the expression of SLC52A2 had a positive association with TMB in 12 cancer types, including ACC, STAD, SARC, PRAD, PAAD, LUSC, LUAD, LGG, KIRC, ESCA, BRCA, BLCA. In contrast, the SLC52A2 expression had a negative association with TMB in COAD (Fig. 4B). Besides, we also used the mRNA sequence data from the TCGA database to assess whether there is an association between SLC52A2 expression and some common immune checkpoint genes. The result revealed that the significant co-expression of SLC52A2 and most immune checkpoint genes were detected in KICH, KIRC, KIRP, LGG, LIHC, and UVM (Fig. 4C).

### Association between SLC52A2 expression with immune cells infiltration in multiple human cancers

It is well known that immune cells play a very important role in the immune microenvironment and can influence the prognosis of cancer patients. Here, we further analyzed the correlation between SLC52A2 expression and infiltration of 6 immune-related cells by the TIMER database. The results showed that SLC52A2 was positively correlated with most immune cell infiltration in KIRP, LGG, and LIHC (Fig. 4D). We further explored the relationship of SLC52A2 with different functional immune infiltrating cells, including T cell (general, CD8^+^, Th1, Th2, Tfh, Th17, Treg, and T cell exhaustion), B cell, monocyte, tumor-assisting macrophages (TAMs), M1 and M2 macrophage, neutrophil, and Dendritic cell. As shown in Fig. 4E, we were excited to observe a strong positive correlation between SLC52A2 and the molecular markers of T cell exhaustion in BLCA, KICH, KIRC, KIRP, LGG, LIHC, PCPG, SARC, TCGT, THCA, THYM, and UVM. Besides, the SLC52A2 and molecular markers of TAMs and M2 macrophages also showed a significant positive correlation in KICH, LIHC, SARC, and UVM. Thus, SLC52A2 may play a crucial role in immune escape and macrophage M2 polarization in the cancers microenvironment. In addition, the correlation between SLC52A2 expression and infiltration of 22 immune cells was analyzed in a variety of tumors according to the CIBERSORT algorithm. Our results are roughly consistent with the above findings (Additional file 8: Fig. S5). For example, the results show that SLC52A2 is positively related to the level of M2 macrophage infiltration in HNSC, LGG, LIHC, SARC, and THYM. We also found that the level of Tregs infiltration, which contributes to immune escape, positively correlated with SLC52A2 expression in BRCA, KIRC, KIRP, LGG, LIHC, LUAD, PAAD, PCPG, PRAD, and STAD (Additional file 8: Fig. S5). It is worth noting that, using the TIDE algorithm, we found that in most tumors, the infiltration level of myeloid-derived suppressor cells is positively associated with the expression of SLC52A2 (Additional file 8: Fig. S5). In summary, we found that SLC52A2 is closely related to the formation of the cancer-promoting immune microenvironment.

### SLC52A2-related gene enrichment analysis

**Fig. 5:**
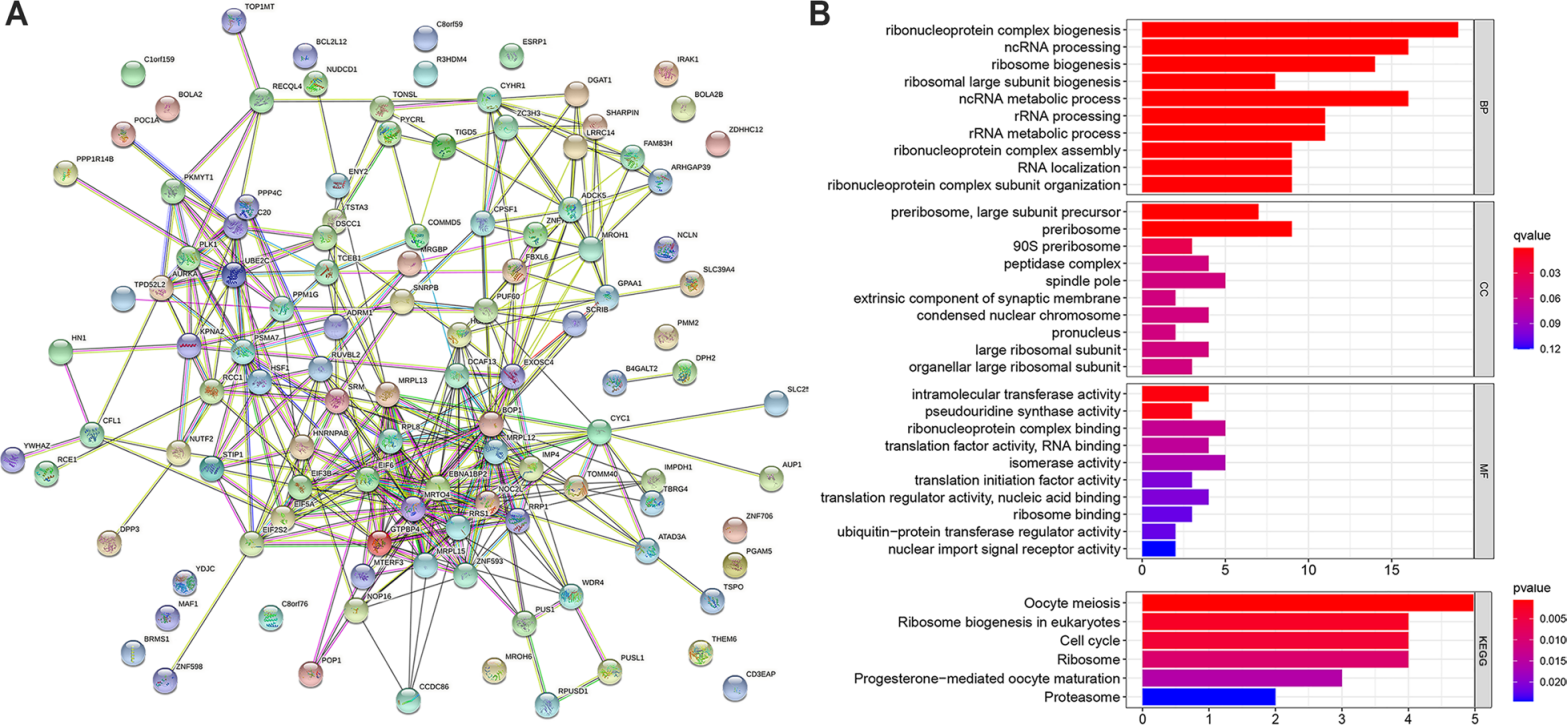
PPI network analysis and KEGG and GO enrichment analysis of SLC52A2-related genes. **A** The PPI network of SLC52A2 is constructed by the GEPIA2 tool and STRING database. **B** The barplot of GO enrichment analysis and KEGG enrichment analysis. *KEGG* Kyoto Encyclopedia of Genes and Genomes, *GO* Gene Ontology, *BP* biological process, *CC* cellular component, *MF* molecular function

To further explore the role of SLC52A2 in tumorigenesis, we used the GEPIA2 tool to obtain 114 genes that were highly correlated with SLC52A2 expression (R > 0.4, Additional file 2: Table S2). Then, the protein interaction network of the above genes was built using the STRING tool (Fig. 5A). We further perform KEGG and GO enrichment analyses. After the screening of GO enrichment analysis, we revealed several enriched gene sets shown in Fig. 5B. The biological process (BP) is mainly enriched in “ribonucleoprotein complex biogenesis”, “ncRNA processing”, “ncRNA metabolic process” and “ribosome biogenesis”. For the result of the cellular component (CC), it was indicated that these genes were mainly related to “preribosome, large subunit precursor” and “preribosome”. Moreover, in the molecular function (MF) analysis, “intramolecular transferase activity” was found to be related to these genes. The KEGG data of Fig. 5B show that “Oocyte meiosis”, “Ribosome biogenesis in eukaryotes”, “Cell cycle” and “Ribosome” might be involved in the effect of SLC52A2 on tumor pathogenesis.

### Analysis of the relationship between SLC52A2 expression and LIHC

**Fig. 6:**
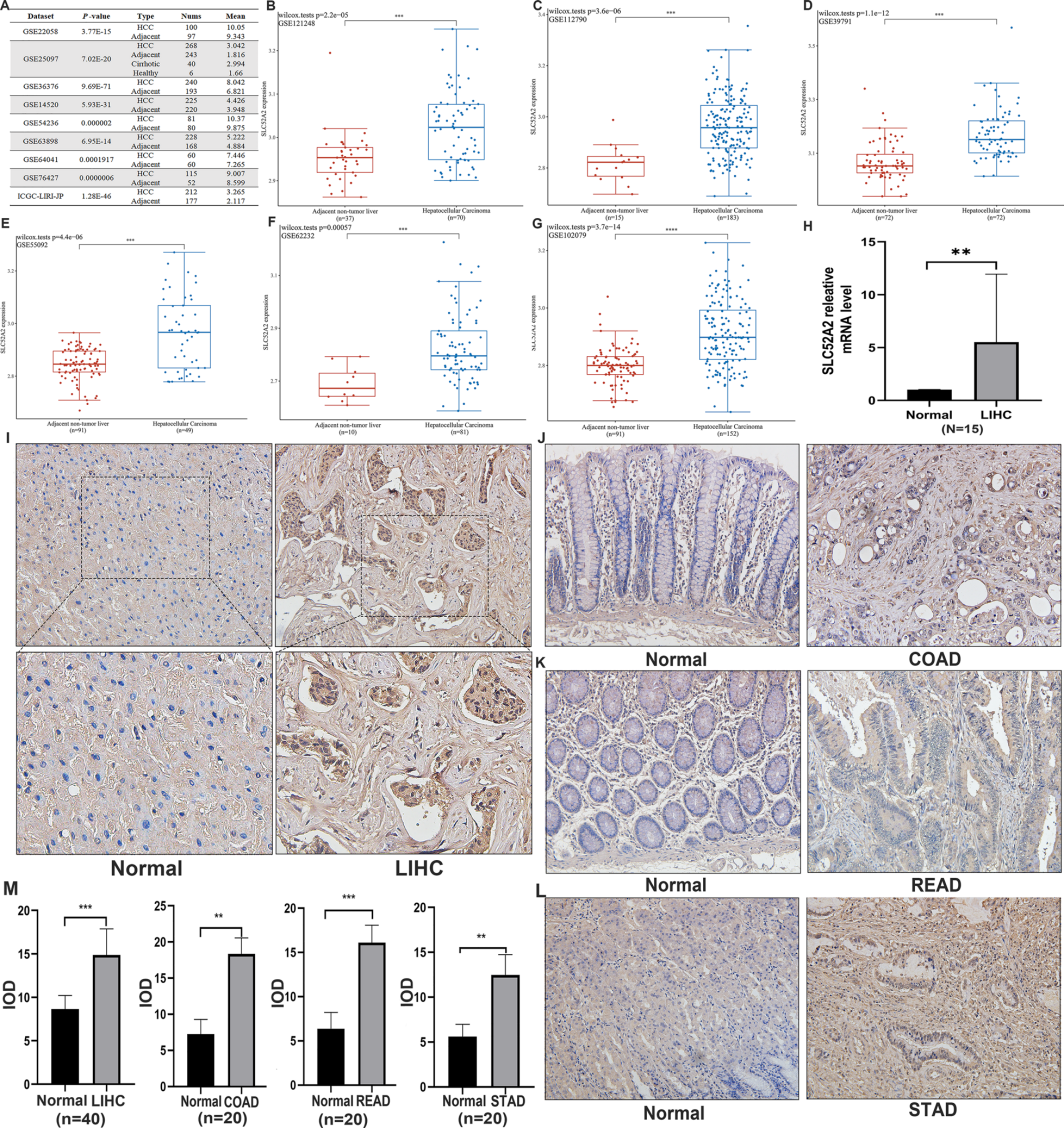
Validation of SLC52A2 expression by HCCDB database (**A**). Validation of SLC52A2 expression by GSE121248 (**B**), GSE102079 (**C**), GSE39791 (**D**), GSE55092 (**E**), GSE62232 (**F**), and GSE112790 (**G**). Differential expression of SLC52A2 in hepatocellular carcinoma and paraneoplastic tissues by qRT-PCR analysis (**H**). Representative images of immunohistochemistry with 200 × and 400 × in LIHC (**I**). Representative images of immunohistochemistry with 200 × in COAD (**J**), READ (**K**), and STAD (**L**). Analysis of the integrated optical density (IOD) values of tumor tissues and paracancerous tissues in LIHC, COAD, READ, and STAD (**M**) (*P < 0.05, **P < 0.01, ***P < 0.001)

From the above analysis, it was found that SLC52A2 was closely related to the prognosis of LIHC. Therefore, we conducted a more in-depth study on the correlation between SLC52A2 and LIHC. We first performed a pairwise difference analysis of LIHC using the TCGA database, and the results continued to show that SLC52A2 expression was much higher in tumors than in paracancerous tissues (Additional file 9: Fig. S6). Next, we validated the expression of SLC52A2 using the HCCDB database (including GSE22058, GSE25097, GSE36376, GSE14520, GSE54236, GSE63898, GSE64041, GSE76427, and ICGC-LIRI-JP datasets), GSE121248, GSE112790, GSE39791, GSE55092, GSE62232, and GSE102079 datasets, and results showed that SLC52A2 was highly expressed in LIHC (Fig. 6A–G). We also verified SLC52A2 mRNA expression by qRT-PCR analysis using hepatocellular carcinoma and paraneoplastic frozen tissues, and the results were consistent with the above (Fig. 6H). Finally, we further evaluated the SLC52A2 expression using IHC. IHC staining showed that SLC52A2 was significantly upregulated in LIHC (Fig. 6I, M; Table 2). Besides, we obtained similar findings in STAD, READ, and COAD (Fig. 6J–M; Additional file 3: Table S3). Based on IHC analysis, 40 patients were divided into a low SLC52A2 expression group (n = 20) and a high SLC52A2 expression group (n = 20). Detailed clinicopathological characteristics in LIHC are summarised in Table 2. Chi-squared analysis revealed that male patients (*P* = 0.034) and patients with larger tumor size (*P* = 0.043), poor tumor differentiation (*P* = 0.025), TNM stage III–IV (*P* = 0.157), vascular invasion (*P* = 0.236) appeared to exhibit the higher expression of SLC52A2.

**Table 2:** The association between SLC52A2 expression and clincopathological features in LIHC

Clinical variables	No. of patients	SLC52A2 expression level		*P* value
	n = 40	Low (n = 20)	High (n = 20)	
Gender				
Male	29	11	18	0.034
Female	11	9	2	
Age (years)				
< 60	31	18	13	0.130
≥ 60	9	2	7	
HBsAg				
Positive	36	18	18	1
Negative	4	2	2	
AFP (ng/ml)				
< 400	16	10	6	0.197
≥ 400	24	10	14	
Liver cirrhosis				
Yes	28	13	15	0.490
No	12	7	5	
Child–Pugh Class				
A	33	18	15	0.405
B	7	2	5	
Tumor size (cm)				
< 5 cm	13	10	3	0.043
≥ 5 cm	27	10	17	
Vascular invasion				
Yes	8	2	6	0.236
No	32	18	14	
Tumor differentiation				
Well	23	15	8	0.025
Poor	17	5	12	
TNM stage				
I–II	29	17	12	0.157
III–IV	11	3	8	

**Fig. 7:**
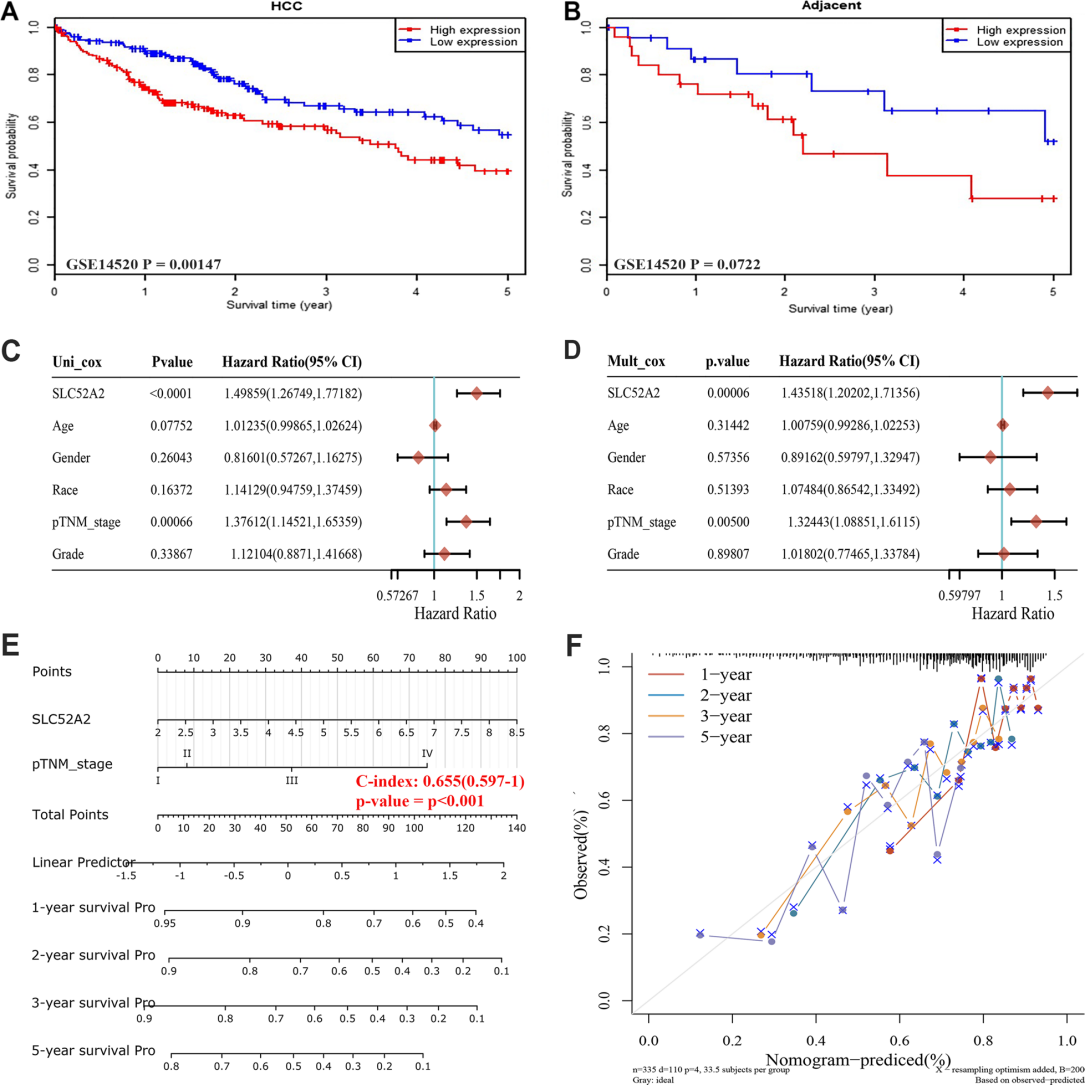
Association between SLC52A2 expression and the prognosis in LIHC. GSE14520 dataset was used to verify the poor prognosis of SLC52A2 in LIHC patients (**A**,** B**).** C**,** D** Hazard ratio and *P*-value of constituents involved in univariate and multivariate Cox regression and some parameters of the SLC52A2 genes [age (years) is a continuous variable; Gender: Male vs Female (reference values); Race: Black/White vs Asian (reference values); pTNM stage: III/IV vs I/II (reference values); Grade: G3/G4 vs G1/G2 (reference values)].** E** Nomogram to predict the 1-year, 2-year, 3-year, and 5-year overall survival of LIHC cancer patients. **F** Calibration curve for the overall survival nomogram model. A dashed diagonal line represents the ideal nomogram, and the red line, blue line, orange line, and purple line represent the 1-year, 2-year, 3-year, and 5-year observed nomograms

We also validated the relationship between SLC52A2 and the prognosis of LIHC patients using the GSE14520 dataset, which showed that high SLC52A2 expression in tumor tissues correlated with poor prognosis of patients (Fig. 7A, B). Therefore, we further explored whether SLC52A2 is an independent prognostic factor for LIHC patients using TCGA data. As seen in Fig. 7C, D, the SLC52A2 expression was significantly associated with OS in LIHC in the univariate and multivariate Cox regression, indicating SLC52A2 expression was an independent predictor of prognosis in LIHC. To establish a clinically applicable method that could predict the survival probability of patients with LIHC, we conducted nomogram predictive models [C-index: 0.655(0.597–1), *P* < 0.001], considering SLC52A2 expression and clinicopathological features, to predict the probability of the 1-, 2-, 3- and 5-year OS (Fig. 7E). The calibration plots for the 1-, 2- and 3-year OS rates were predicted well compared with an ideal model (Fig. 7F). In addition to LIHC, we found SLC52A2 to be an independent risk factor in CESC, KIRC, and KIRP (Additional file 10: Fig. S7).

Finally, to further validate the reliability of the above SLC52A2 enrichment analysis results and to further explore the role of SLC52A2 in the progression of LIHC, we divided the patients into high and low expression groups according to their median SLC52A2 expression levels and performed KEGG and GO enrichment analysis. The results were consistent with the above analysis (Additional file 11: Fig. S8). For example, compared with patients with low SLC52A2 expression, patients with high SLC52A2 expression are significantly enriched in the “Oocyte meiosis”, “Cell cycle” and “Ribosome” pathways (Additional file 11: Fig. S8).

### Prediction and analysis of upstream lncRNA and miRNAs of SLC52A2 in LIHC

**Fig. 8:**
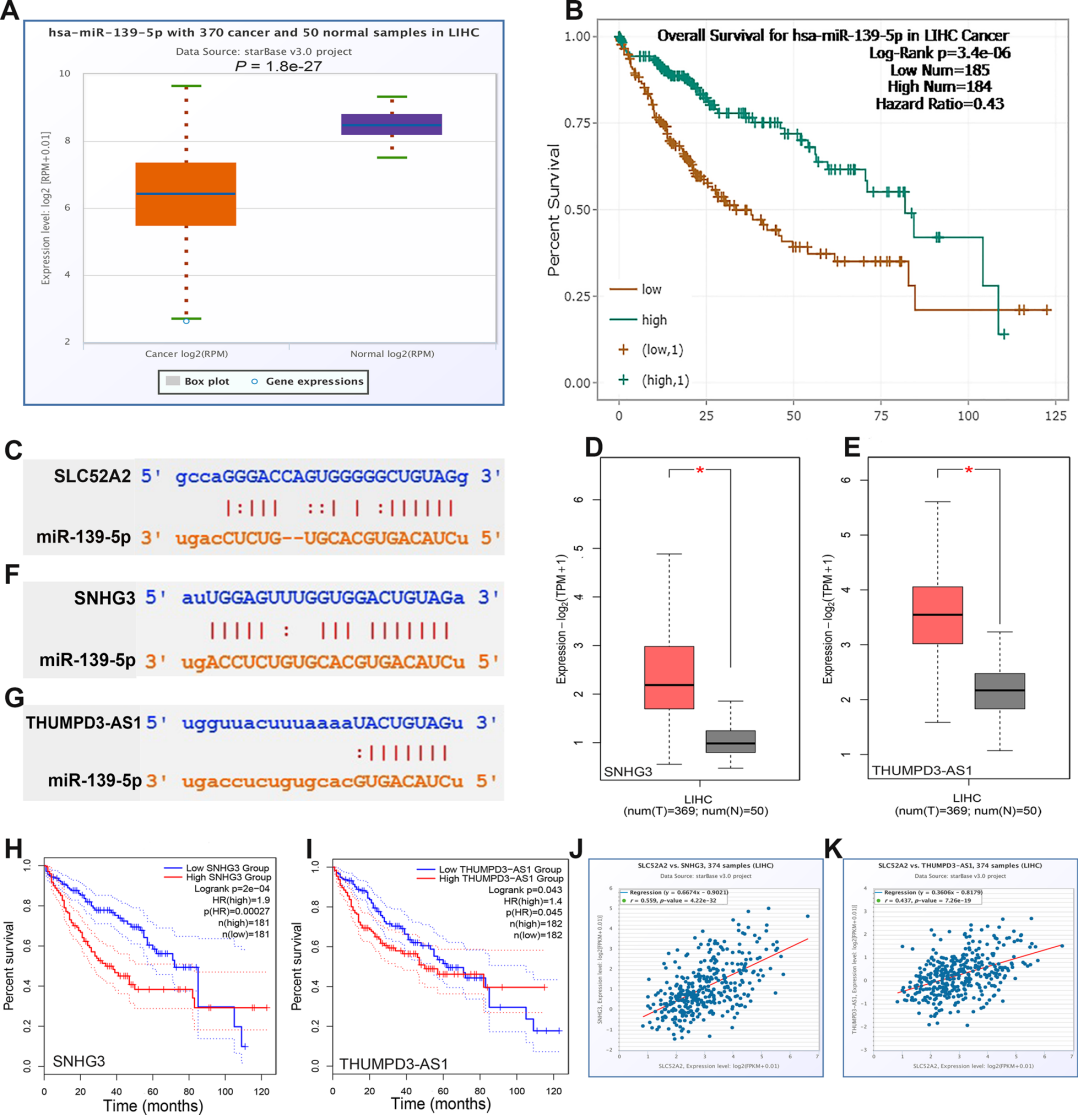
Prediction and analysis of upstream lncRNA and miRNAs of SLC52A2 in LIHC. **A** Analysis of hsa-miR-139-5p expression in hepatocellular carcinoma and paraneoplastic tissues using starbase database. **B** Correlation of hsa-miR-139-5p with prognosis using the starbase database. **C** The matching analysis of related sequences of SLC52A2 and has-miR-139-5p. **D**, **E** Analysis of SNHG3 and THUMPD3-AS1 expression in hepatocellular carcinoma and paraneoplastic tissues using the GEPIA2. **F**, **G** The matching analysis of related sequences of SNHG3, THUMPD3-AS1, and has-miR-139-5p. **H**, **I** Correlation of SNHG3 and THUMPD3-AS1 with prognosis using the GEPIA2. **J**, **K** The SLC52A2 and SNHG3, THUMPD3-AS1 co-expression analysis using the starbase database (*P < 0.05)

It is well known that non-coding RNA (ncRNA) is responsible for regulating gene expression. To determine whether SLC52A2 is regulated by certain ncRNAs, we first predicted upstream miRNAs that may bind to SLC52A2 using the starBase database and finally identified 13 miRNAs that were significantly associated (Additional file 12: Fig. S9A). However, based on the action mechanism of miRNAs in regulating target gene expression, there should be a negative association between miRNAs and SLC52A2. Therefore, we considered hsa-miR-122-5p (*R* = − 0.473) and hsa-miR-139-5p (*R* = − 0.449), which have the largest negative correlation coefficients, maybe the upstream miRNA of SLC52A2. As shown in Fig. 8A, B, hsa-miR-139-5p was expressed at much lower levels in tumors, and low expression was closely associated with poor prognosis. Figure 8C shows a match analysis of the SLC52A2 sequences and has-miR-139-5p sequences using the StarBase database. Although the expression of hsa-miR-122-5p was also lower in cancer, and prognostic analysis found that its high expression was associated with poor prognosis (Additional file 12: Fig. S9B, C), which contradicted the relationship between SLC52A2 and prognosis. Thus, we speculate that hsa-miR-139-5p is the most likely upstream miRNA of SLC52A2.

Next, the starBase database was used to predict the upstream lncRNAs of hsa-miR-139-5p. A total of 29 possible lncRNAs were predicted (Additional file 12: Fig. S9D). The results showed that the largest negative correlation coefficients with hsa-miR-139-5p were for SNHG3 (*R* = − 0.443) and THUMPD3-AS1 (*R* = − 0.362). Based on the competing endogenous RNA (ceRNA) hypothesis, lncRNA could rise the expression of mRNA by competitively binding to shared miRNAs. Thus, there should be a positive correlation between lncRNA and mRNA or a negative relationship between lncRNA and miRNA. As shown in Fig. 8D–I, we found that SNHG3 and THUMPD3-AS1 were more highly expressed in LIHC and were associated with poor prognosis by GEPIA2. Besides, we also found that SLC52A2 expression was positively correlated with SNHG3 (*R* = 0.559) and THUMPD3-AS1 (*R* = 0.437) using the starBase database (Fig. 8J, K). Taking expression analysis, survival analysis, and correlation analysis into consideration, SNHG3 and THUMPD3-AS1 might be the two most potential upstream lncRNAs of the hsa-miR-139-5p-SLC52A2 axis in LIHC.

## Discussion

Despite the close association of SLC52A2 with human health, little data is available on this transporter, especially in tumors. In this study, we systematically analyzed the association of SLC52A2 with multiple human tumor types. We found that of the 28 tumors whose normal tissues could acquire through the ONCOMINE, GTEx, and TCGA databases, 27 of them were highly expressed, and SLC52A2 expression increased with increasing tumor stage in multiple tumors. As previous studies have demonstrated that riboflavin in the blood of tumor patients is reduced [20, 21]. Thus, we hypothesize that the rapid metabolism and proliferation of tumor cells increases the rate of riboflavin consumption and uptake, which in turn induces the SLC52A2 expression in the organism to facilitate the supply of cytosolic riboflavin.

To explore whether the currently published articles on SLC52A2 expression and cancer are consistent with our conclusions, we searched PubMed, Embase, the Cochrane Library, on April 30, 2021, using the following terms in [All Fields]: BVVLS2, GPR172A, SLC52A2, HRFT3, GPCR41, RFVT2, RFT3, BVVLS2. We retrieved three relevant studies, and all were consistent with our findings from publicly available databases. For example, by analyzing 80 paired gastric cancer and corresponding adjacent non-cancerous tissues in the GSE27342 dataset, Cheng et al. found that the SLC52A2 expression was elevated 1.7-fold in gastric cancer, and the difference was statistically significant [10]. Similarly, Vathipadiekal found the significant upregulation of SLC52A2 in ovarian cancer tissues by using public data (*P* < 0.001). And, the above findings were then confirmed using the GSE26712 dataset. Also, they compared serum SLC52A2 levels in patients with advanced ovarian cancer and normal age-matched controls by enzyme-linked immunosorbent assay. The results showed a 2.9-fold increase in the patients with ovarian cancer [22]. Recently, Tutino et al. estimated the mRNA levels of SLC52A2 in tumor tissues and surrounding normal mucosa of 24 colorectal cancer patients. They found that SLC52A2 expression was increased in tumor tissues (*P* < 0.001). Notably, they also found that SLC52A2 protein levels were decreased in tumors as compared to normal mucosa, contrary to their results at the SLC52A2 mRNA level. We showed by immunohistochemical analysis that SLC52A2 was highly expressed in both COAD and READ [23].

We also found that high SLC52A2 expression was a risk factor and related to poor OS, DSS, and PFI. Interestingly, we also revealed that patients with cancer harboring SLC52A2 mutations had a worse prognosis. Besides, the SLC52A2 expression has good diagnostic value for multiple tumors. We then selected LIHC that were associated with all of these indicators for in-depth analysis, and we found that SLC52A2 expression was an independent prognostic indicator for patients with LIHC. Recently, it was found that the expression of SNHG3 was significantly higher in hepatocellular carcinoma than in paracancerous tissues [24]. and the overexpression of SNHG3 can promote cell invasion and epithelial-mesenchymal transition in liver cancer [25]. Wu et al*. *[26] revealed that hsa-miR-139-5p expression was reduced in liver cancer compared to normal liver tissues and that low miR-139-5p expression was associated with a poorer prognosis. Mechanistic studies suggested that low miR-139-5p expression can increase the invasive and proliferative capacity of liver cancer cells. In the current study, we demonstrated that SNHG3 and THUMPD3-AS1/hsa-miR-139-5p-SLC52A2 axis were identified as potential regulatory pathways in LIHC.

Immune checkpoints are a kind of suppressor molecule that acts as a beneficial effect in the human immune system, preventing inflammatory damage caused by the over-activation of T cells [27]. However, tumor cells can escape immune surveillance and achieve immuno-escape by overexpressing checkpoint molecules to suppress the anti-tumor response of the immune system [28, 29]. Our results show that SLC52A2 positively correlates with the expression of multiple immune checkpoint genes in pan-cancer, which is consistent with the conclusion that high SLC52A2 expression is related to a poor prognosis. The ICIs can prevent tumor progression by blocking the interaction of immune checkpoints and their ligands, disrupting immune tolerance, and promoting the clearance of tumor cells. A major advance in cancer therapy in recent years has been the development of ICIs, which have produced durable responses and improved survival in many kinds of solid malignancies [30–32]. However, the majority of patients treated with ICIs do not benefit and there is an urgent need to explore accurate predictive biomarkers of ICIs response to use them more selectively. TMB refers to the total number of somatic mutations per megabase of the interrogated genomic sequence, which varies across malignancies [33]. Increasing evidence indicates that the TMB may be a predictive biomarker for immunotherapy in a variety of solid tumors [34–36]. The higher the TMB of the cancer patients, the better the response to the ICI [37] and the longer the OS [38]. MSI was defined as any change in microsatellite length due to insertion or deletion of repetitive units in tumor tissues, with the emergence of new microsatellite DNA alleles. Similarly, MSI can also be used to guide decisions about adjuvant chemotherapy, targeted therapy, and immunotherapy for tumors [39, 40]. Tumors with microsatellite instability-high were found to be sensitive to immune checkpoint blockade (ICB) [41]. Our study showed that the expression of SLC52A2 was positively associated with TBM and MSI in most tumor types. Therefore, tumor patients with high SLC52A2 expression may have a higher response rate to ICIs.

Immune infiltration in the tumor microenvironment plays a crucial role in tumor development and will affect the clinical prognosis of tumor patients [42]. We demonstrated that SLC52A2 is positively associated with most immune cell infiltration and immune marker sets of immune cells, such as TAMs, M2 macrophages, and T cell exhaustion in several tumors, including LIHC. As the most abundant innate immune cells in the tumor microenvironment, various studies have shown that TAMs can enhance tumorigenesis and progression by promoting angiogenesis in tumor tissues, strengthening the metastatic invasion of tumor cells, and suppressing anti-tumor immunity [43, 44]. As we know, M2 macrophages also have a role in promoting tumor growth, invasion, and metastasis [45, 46]. Since TAMs possess most of the representative properties of M2 macrophages, TAMs are often considered to be M2 macrophages [47, 48]. T cell exhaustion refers to cancer patients where T-cells are continuously provoked by antigens, cellular memory does not differentiate effectively and T cells gradually become exhausted. It is one of the main factors of immune dysfunction in cancer patients. Thus, SLC52A2 may be involved in the progression of tumors such as LIHC by potentially regulating M2 cell polarisation and inducing T cell depletion.

Finally, the KEGG and GO enrichment analysis disclosed that SLC52A2 was mainly enriched in oocyte meiosis, ribosome biogenesis in eukaryotes, and cell cycle. It is well known that the most important feature of cancer cells is their abnormal cell cycle activity and unlimited replication potential. Thus, the above pathway enriched by SLC52A2 may promote cancer progression.

Notably, although in this article we have conducted a comprehensive analysis of SLC52A2 in various tumors, there are still some limitations. This study is based on bioinformatics analysis and only validated the expression of SLC52A2 in LIHC, STAD, COAD, and READ at the tissue level, while the role of SLC52A2 in other tumors requires further experiments to confirm our results.

## Conclusion

**Fig. 9:**
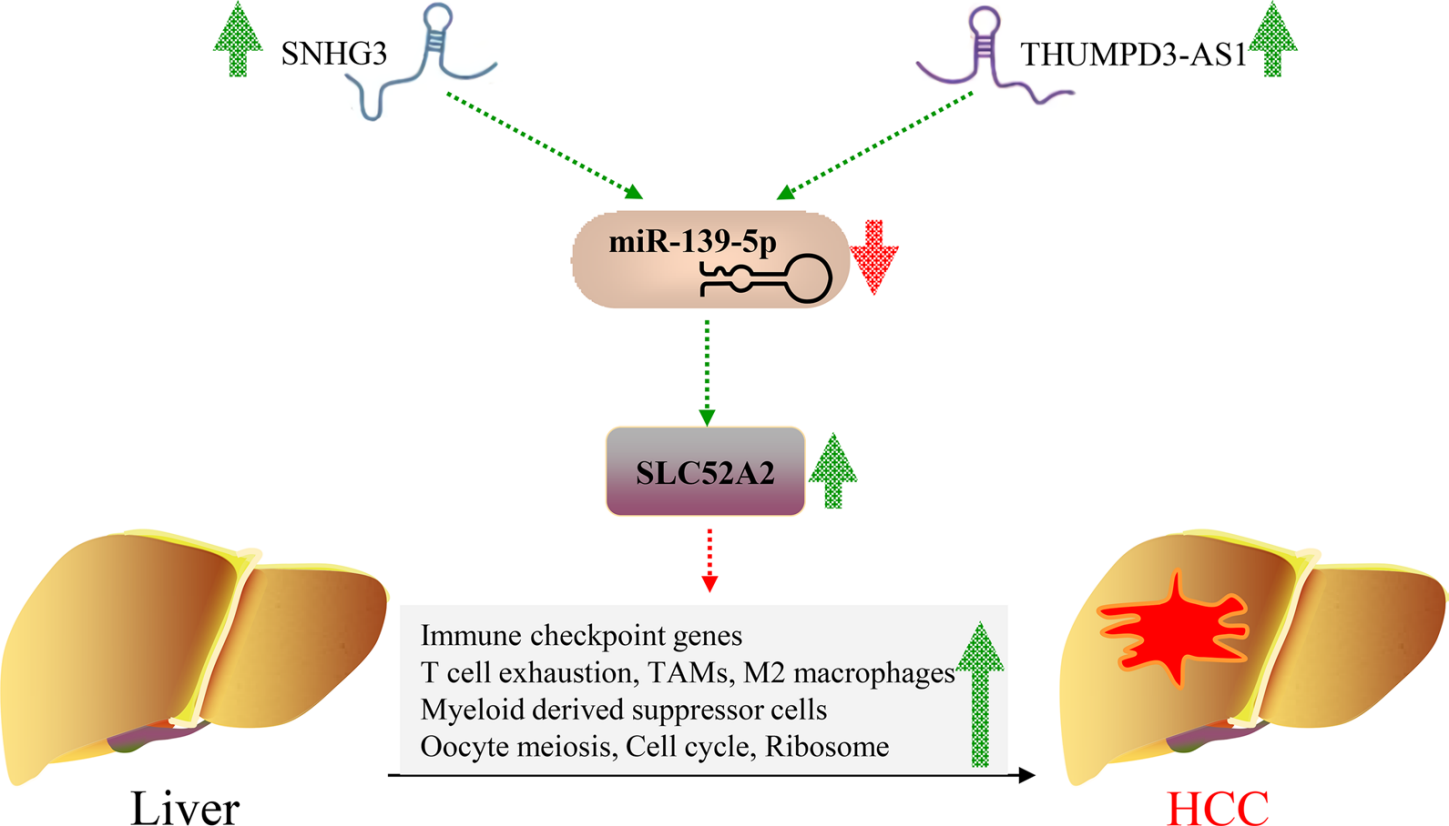
Pattern diagram of SLC52A2 promoting hepatocellular carcinoma. Green spikes represent up-regulated expression in tumor tissue, red spikes represent down-regulated expression in tumor tissue. *TAMs* tumor-assisting macrophages

In conclusion, our study demonstrated that SLC52A2 was highly expressed in almost all tumors and associated with poor prognosis, diagnosis, mutations, TMB, MSI, common immune checkpoint genes, and immune cells infiltration in most cancers (Fig. 9). It may promote cancer progression through its involvement in oocyte meiosis, eukaryotic ribosome biogenesis, and cell cycle. Besides, the SLC52A2 expression was an independent prognostic factor for LIHC, and SNHG3 and THUMPD3-AS1/hsa-miR-139-5p-SLC52A2 axis may be the potential regulatory pathways in LIHC (Fig. 9).

## Supplementary Information

**Additional file 1: Table S1.** Database and its website.

**Additional file 2: Table S2.** 114 genes highly correlated with SLC52A2 expression using GEPIA2 (R > 0.4).

**Additional file 3: Table S3.** The clinicopathological details with STAD, COAD, and READ.

**Additional file 4: Fig. S1.** mRNA expression of SLC52A2 gene in normal human tissues (A). SLC52A2 mRNA expression in 38 kinds of tumor cell lines from the CCLE database (B). (^**^P < 0.01).

**Additional file 5: Fig. S2.** SLC52A2 mRNA expression in different immune subtypes in BRCA, KIRC, KIRP, LGG, LIHC, LUAD, LUSC, OV, PAAD, PRAD, SARC, STAD, and TGCT.

**Additional file 6: Fig. S3.** The AUC of the ROC in BRCA, COAD, HNSC, KIRC, LIHC, LUAD, LUSC, PRAD, and THCA.

**Additional file 7: Fig. S4.** SLC52A2 mutation landscape. (A) SLC52A mutation level from the cBioPortal database. (B) Mutation diagram of SLC52A2 in different cancer types across protein domains from the cBioPortal database. (C) Pie chart showing the percentage of the different mutation types of SLC52A2 in cancers according to the COSMIC database. (D-G) The genetic alteration of SLC52A2 and the survival prognosis of cancers by the cBioPortal database.

**Additional file 8: Fig. S5.** the correlation between SLC52A2 expression and immune cell infiltration using CIBERSORT and TIDE algorithms.

**Additional file 9: Fig. S6.** Pairwise difference analysis of SLC52A2 expression in the TCGA-LIHC cohort.

**Additional file 10: Fig. S7.** Hazard ratio and *P*-value of constituents involved in univariate and multivariate Cox regression and some parameters of the SLC52A2 genes in CESC(A), KIRC (B), and KIRP(C). [age (years) is a continuous variable; Gender: Male vs Female (reference values); Race: Black/White vs Asian (reference values); pTNM stage: III/IV vs I/II (reference values); Grade: G3/G4 vs G1/G2 (reference values)].

**Additional file 11: Fig. S8.** KEGG and GEO enrichment analysis in the LIHC-TCGA cohort. KEGG, Kyoto Encyclopedia of Genes and Genomes; GO, Gene Ontology; BP, biological process; CC, cellular component.

**Additional file 12: Fig. S9.** Prediction and analysis of upstream lncRNA and miRNAs of SLC52A2 in Hepatocellular carcinoma. (A) Correlation analysis of SLC52A2 and miRNA using starbase database. (B) Analysis of hsa-miR-122-5p expression in hepatocellular carcinoma and paraneoplastic tissue using starbase database. (C) Correlation of hsa-miR-122-5p with prognosis using the starbase database. (D) Correlation analysis of hsa-miR-139-5p and lncRNA using starbase database.

## Data Availability

The data used to support the findings of this study are available from the corresponding author upon request.
