# Supplementary material for: Systematic analysis of the role of SLC52A2 in multiple human cancers
Source: Cancer Cell Int. 2022 Jan 6;22:8. doi: 10.1186/s12935-021-02432-7 (PMC8739691; doi:10.1186/s12935-021-02432-7)
Supplement: Supplementary file 1 — Additional file 1: Table S1. Database and its website. [file 12935_2021_2432_MOESM1_ESM.docx]

| Table S1 Database and its website |  |
| --- | --- |
| Databases | Website |
| Xena Browser | https://xenabrowser.net/datapages/ |
| The Cancer Cell Line Encyclopedia | https://portals.broadinstitute.org/ccle/about |
| ONCOMINE | https://www.oncomine.org/resource/main.html |
| TISIDB | http://cis.hku.hk/TISIDB/ |
| cBioPortal | http://www.cbioportal.org/ |
| Catalogue of Somatic Mutations in Cancer | https://cancer.sanger.ac.uk/cosmic/ |
| Gene Expression Omnibus | https://www.ncbi.nlm.nih.gov/geo/ |
| Kaplan-Meier Plotter | https://kmplot.com/analysis/ |
| GEPIA2 | http://gepia2.cancer-pku.cn/#index |
| STRING | https://www.string-db.org/ |
| Gene Set Enrichment Analysis | https://www.gsea-msigdb.org/gsea/downloads.jsp |
| TIMER | https://cistrome.shinyapps.io/timer/ |
| Sangerbox | http://sangerbox.com/ |
| The Cancer Genome Atlas Program | https://www.cancer.gov/about-nci/organization/ccg/research/structural-genomics/tcga |
| Starbase  Integrative Molecular Database of Hepatocellular Carcinoma | https://starbase.sysu.edu.cn/  http://lifeome.net/database/hccdb/home.html |
| TIMER2.0 | http://timer.cistrome.org/ |
