# Supplementary material for: Systematic analysis of the role of SLC52A2 in multiple human cancers
Source: Cancer Cell Int. 2022 Jan 6;22:8. doi: 10.1186/s12935-021-02432-7 (PMC8739691; doi:10.1186/s12935-021-02432-7)
Supplement: Supplementary file 2 — Additional file 2: Table S2. 114 genes highly correlated with SLC52A2 expression using GEPIA2 (R > 0.4). [file 12935_2021_2432_MOESM2_ESM.docx]

| **Table S2** 114 genes highly correlated with SLC52A2 expression using GEPIA2 (R > 0.4) | | |
| --- | --- | --- |
| Gene Symbol | Gene ID | PCC |
| FBXL6 | ENSG00000182325.10 | 0.73 |
| HGH1 | ENSG00000235173.6 | 0.68 |
| BOP1 | ENSG00000261236.5 | 0.66 |
| ADCK5 | ENSG00000173137.11 | 0.63 |
| HSF1 | ENSG00000185122.10 | 0.63 |
| SHARPIN | ENSG00000179526.16 | 0.62 |
| EXOSC4 | ENSG00000178896.6 | 0.61 |
| PYCRL | ENSG00000104524.13 | 0.61 |
| SCRIB | ENSG00000180900.16 | 0.6 |
| TSTA3 | ENSG00000104522.15 | 0.59 |
| MAF1 | ENSG00000179632.9 | 0.58 |
| COMMD5 | ENSG00000170619.9 | 0.58 |
| RRS1 | ENSG00000179041.3 | 0.56 |
| SLC39A4 | ENSG00000147804.9 | 0.55 |
| TOP1MT | ENSG00000184428.12 | 0.54 |
| RECQL4 | ENSG00000160957.12 | 0.52 |
| FAM83H | ENSG00000180921.6 | 0.52 |
| TONSL | ENSG00000160949.16 | 0.52 |
| PUF60 | ENSG00000179950.13 | 0.52 |
| ADRM1 | ENSG00000130706.12 | 0.51 |
| DCAF13 | ENSG00000164934.13 | 0.51 |
| PUSL1 | ENSG00000169972.11 | 0.51 |
| RPL8 | ENSG00000161016.15 | 0.51 |
| ZNF707 | ENSG00000181135.15 | 0.51 |
| DGAT1 | ENSG00000185000.9 | 0.5 |
| ZC3H3 | ENSG00000014164.6 | 0.5 |
| GPAA1 | ENSG00000197858.10 | 0.49 |
| CYC1 | ENSG00000179091.4 | 0.49 |
| PPP1R14B | ENSG00000173457.10 | 0.49 |
| ATAD3A | ENSG00000197785.13 | 0.48 |
| TCEB1 | ENSG00000154582.16 | 0.48 |
| NOC2L | ENSG00000188976.10 | 0.48 |
| RPUSD1 | ENSG00000007376.7 | 0.48 |
| LRRC14 | ENSG00000160959.7 | 0.47 |
| CFL1 | ENSG00000172757.12 | 0.46 |
| NOP16 | ENSG00000048162.19 | 0.46 |
| MRPL15 | ENSG00000137547.8 | 0.46 |
| EIF6 | ENSG00000242372.6 | 0.46 |
| BOLA2B | ENSG00000169627.7 | 0.46 |
| TOMM40 | ENSG00000130204.12 | 0.46 |
| NUDCD1 | ENSG00000120526.10 | 0.46 |
| ESRP1 | ENSG00000104413.15 | 0.46 |
| ENY2 | ENSG00000120533.12 | 0.45 |
| CPSF1 | ENSG00000071894.14 | 0.45 |
| NCLN | ENSG00000125912.10 | 0.45 |
| AUP1 | ENSG00000115307.16 | 0.45 |
| MRTO4 | ENSG00000053372.4 | 0.45 |
| SNRPB | ENSG00000125835.17 | 0.44 |
| MROH6 | ENSG00000204839.8 | 0.44 |
| IMPDH1 | ENSG00000106348.16 | 0.44 |
| UBE2C | ENSG00000175063.16 | 0.44 |
| C8orf76 | ENSG00000189376.11 | 0.44 |
| MRGBP | ENSG00000101189.6 | 0.44 |
| STIP1 | ENSG00000168439.16 | 0.44 |
| ZNF593 | ENSG00000142684.7 | 0.44 |
| WDR4 | ENSG00000160193.11 | 0.44 |
| THEM6 | ENSG00000130193.7 | 0.43 |
| PPP4C | ENSG00000149923.13 | 0.43 |
| PSMA7 | ENSG00000101182.14 | 0.43 |
| EIF5A | ENSG00000132507.17 | 0.43 |
| PUS1 | ENSG00000177192.13 | 0.43 |
| ZNF706 | ENSG00000120963.11 | 0.43 |
| BCL2L12 | ENSG00000126453.9 | 0.43 |
| TBRG4 | ENSG00000136270.13 | 0.43 |
| MRPL13 | ENSG00000172172.7 | 0.43 |
| YWHAZ | ENSG00000164924.17 | 0.43 |
| RCE1 | ENSG00000173653.7 | 0.42 |
| MTERF3 | ENSG00000156469.8 | 0.42 |
| HN1 | ENSG00000189159.15 | 0.42 |
| TIGD5 | ENSG00000179886.5 | 0.42 |
| DSCC1 | ENSG00000136982.5 | 0.42 |
| CDC20 | ENSG00000117399.13 | 0.42 |
| POC1A | ENSG00000164087.7 | 0.42 |
| PMM2 | ENSG00000140650.11 | 0.42 |
| MRPL12 | ENSG00000262814.6 | 0.42 |
| PLK1 | ENSG00000166851.14 | 0.42 |
| MROH1 | ENSG00000179832.17 | 0.42 |
| DPP3 | ENSG00000254986.7 | 0.42 |
| ZDHHC12 | ENSG00000160446.18 | 0.42 |
| AURKA | ENSG00000087586.17 | 0.42 |
| R3HDM4 | ENSG00000198858.9 | 0.42 |
| NUTF2 | ENSG00000102898.11 | 0.42 |
| FAM83H-AS1 | ENSG00000203499.10 | 0.41 |
| BOLA2 | ENSG00000183336.7 | 0.41 |
| ARHGAP39 | ENSG00000147799.11 | 0.41 |
| EBNA1BP2 | ENSG00000117395.10 | 0.41 |
| CYHR1 | ENSG00000187954.12 | 0.41 |
| RCC1 | ENSG00000180198.15 | 0.41 |
| GTPBP4 | ENSG00000107937.18 | 0.41 |
| SLC25A39 | ENSG00000013306.15 | 0.41 |
| YDJC | ENSG00000161179.13 | 0.41 |
| TSPO | ENSG00000100300.17 | 0.41 |
| IMP4 | ENSG00000136718.9 | 0.41 |
| C1orf159 | ENSG00000131591.17 | 0.41 |
| EIF3B | ENSG00000106263.17 | 0.41 |
| PPM1G | ENSG00000115241.10 | 0.41 |
| IRAK1 | ENSG00000184216.11 | 0.4 |
| CD3EAP | ENSG00000117877.10 | 0.4 |
| B4GALT2 | ENSG00000117411.16 | 0.4 |
| ZNF598 | ENSG00000167962.12 | 0.4 |
| TPD52L2 | ENSG00000101150.17 | 0.4 |
| SRM | ENSG00000116649.9 | 0.4 |
| PKMYT1 | ENSG00000127564.16 | 0.4 |
| RRP1 | ENSG00000160214.12 | 0.4 |
| DPH2 | ENSG00000132768.13 | 0.4 |
| HNRNPAB | ENSG00000197451.10 | 0.4 |
| C8orf59 | ENSG00000176731.11 | 0.4 |
| CCDC86 | ENSG00000110104.11 | 0.4 |
| KPNA2 | ENSG00000182481.8 | 0.4 |
| BRMS1 | ENSG00000174744.13 | 0.4 |
| PGAM5 | ENSG00000247077.6 | 0.4 |
| EIF2S2 | ENSG00000125977.6 | 0.4 |
| POP1 | ENSG00000104356.10 | 0.4 |
| RUVBL2 | ENSG00000183207.12 | 0.4 |
