# Supplementary material for: Systematic analysis of the role of SLC52A2 in multiple human cancers
Source: Cancer Cell Int. 2022 Jan 6;22:8. doi: 10.1186/s12935-021-02432-7 (PMC8739691; doi:10.1186/s12935-021-02432-7)
Supplement: Supplementary file 3 — Additional file 3: Table S3. The clinicopathological details with STAD, COAD, and READ. [file 12935_2021_2432_MOESM3_ESM.docx]

| **Table S3** The clinicopathological details with STAD, COAD, and READ | | | |
| --- | --- | --- | --- |
| **Clinical variables** | **STAD**  **(n=20)** | **COAD**  **(n=20)** | **READ**  **(n=20)** |
|  |  |  |  |
| Gender |  |  |  |
| Male | 8 | 13 | 6 |
| Female | 12 | 7 | 14 |
| Age (years) |  |  |  |
| <60 | 14 | 12 | 13 |
| ≥60 | 6 | 8 | 7 |
| Tumor size (cm) |  |  |  |
| <5cm | 13 | 9 | 12 |
| ≥5cm | 7 | 11 | 8 |
| Vascular invasion |  |  |  |
| Yes | 4 | 6 | 5 |
| NO | 16 | 14 | 15 |
| Nerve violations |  |  |  |
| Yes | 5 | 7 | 6 |
| NO | 15 | 13 | 14 |
| Tumor differentiation |  |  |  |
| Well | 8 | 11 | 7 |
| Poor | 12 | 9 | 13 |
| TNM stage |  |  |  |
| I-II | 13 | 11 | 12 |
| III-IV | 7 | 9 | 8 |
