# Supplementary figures and images for: Systematic analysis of the role of SLC52A2 in multiple human cancers
Source: Cancer Cell Int. 2022 Jan 6;22:8. doi: 10.1186/s12935-021-02432-7 (PMC8739691; doi:10.1186/s12935-021-02432-7)

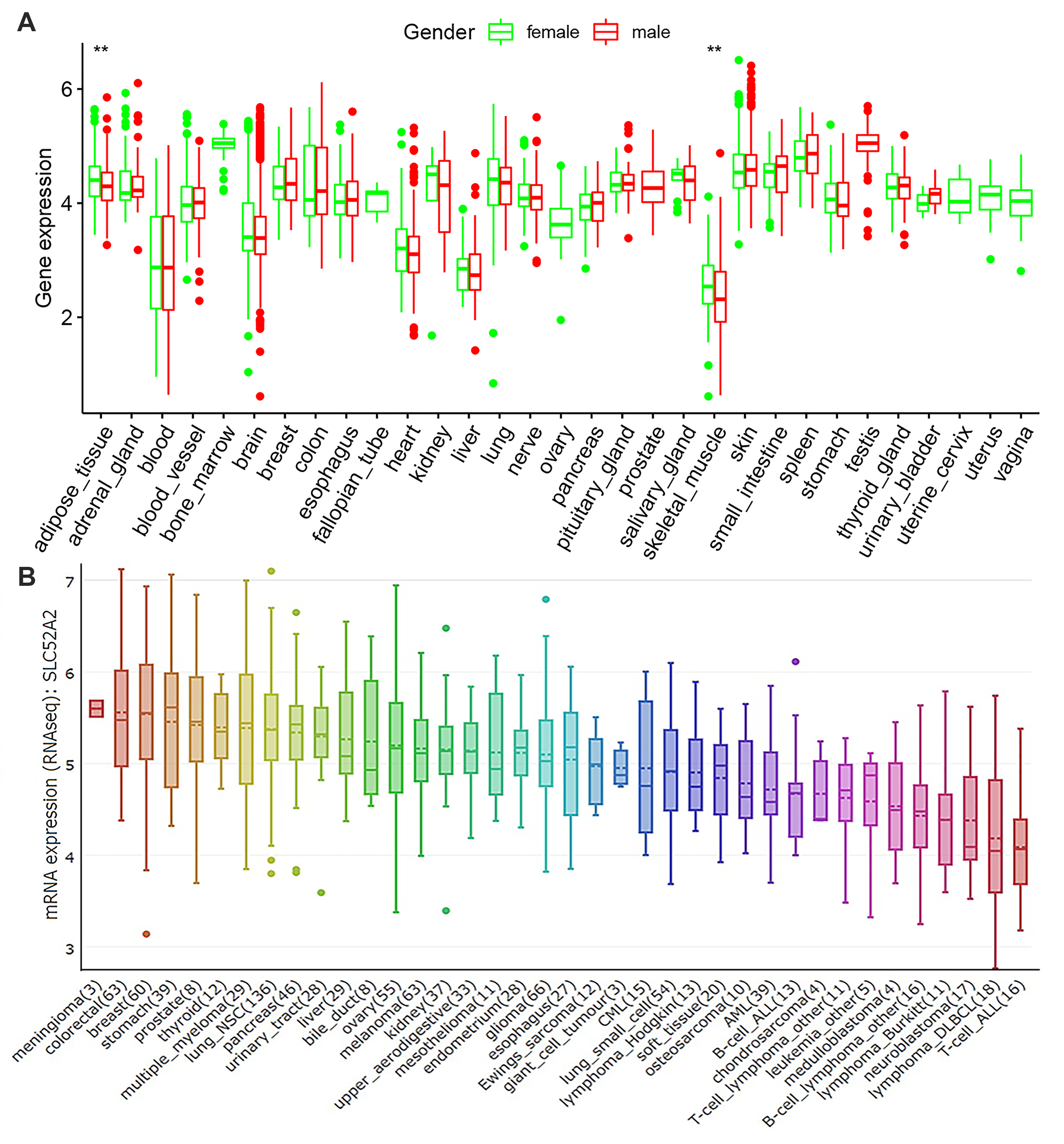

Supplement: Supplementary file 4 — Additional file 4: Fig. S1. mRNA expression of SLC52A2 gene in normal human tissues (A). SLC52A2 mRNA expression in 38 kinds of tumor cell lines from the CCLE database (B). (**P < 0.01). [file 12935_2021_2432_MOESM4_ESM.tif]

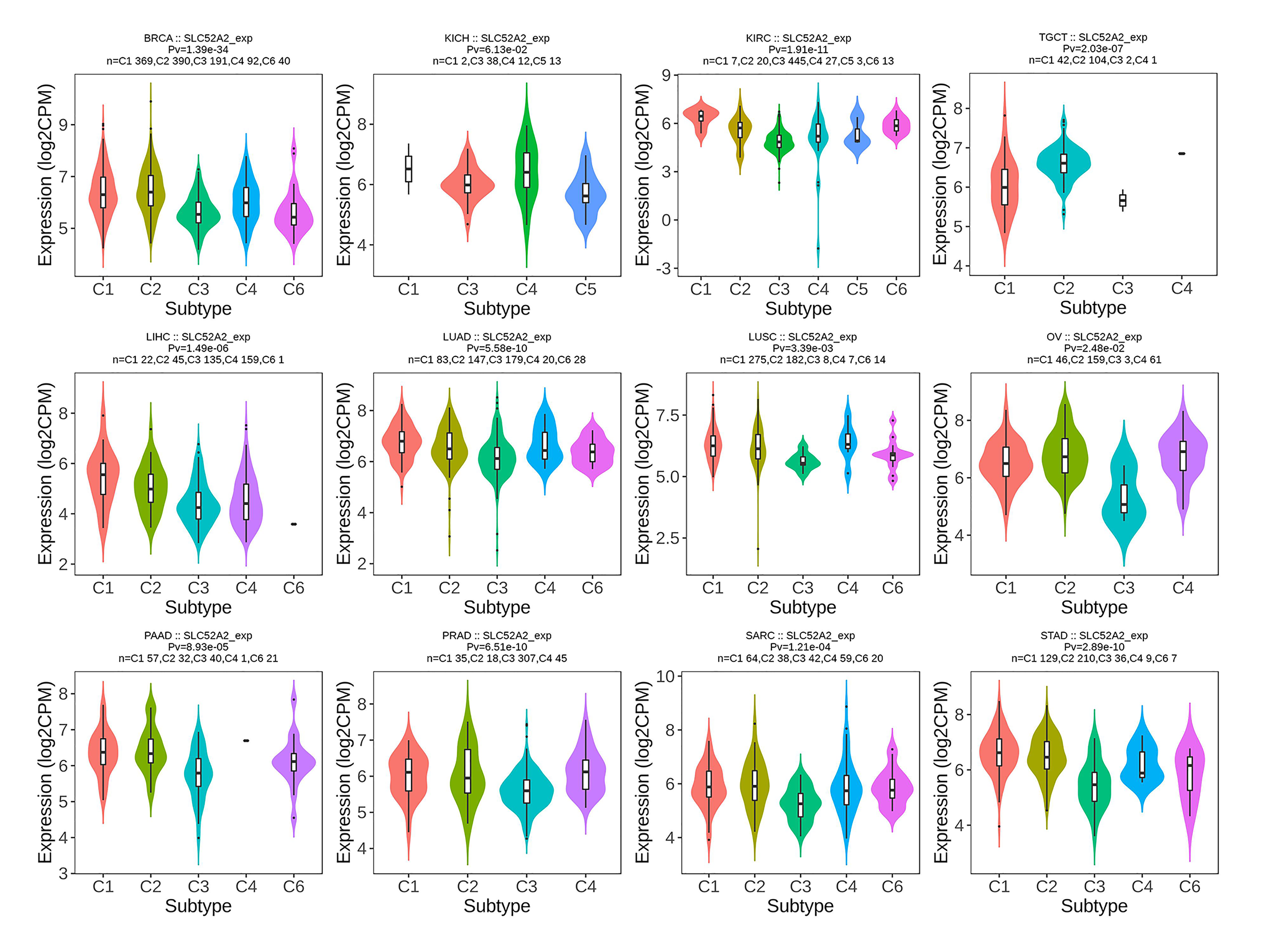

Supplement: Supplementary file 5 — Additional file 5: Fig. S2. SLC52A2 mRNA expression in different immune subtypes in BRCA, KIRC, KIRP, LGG, LIHC, LUAD, LUSC, OV, PAAD, PRAD, SARC, STAD, and TGCT. [file 12935_2021_2432_MOESM5_ESM.tif]

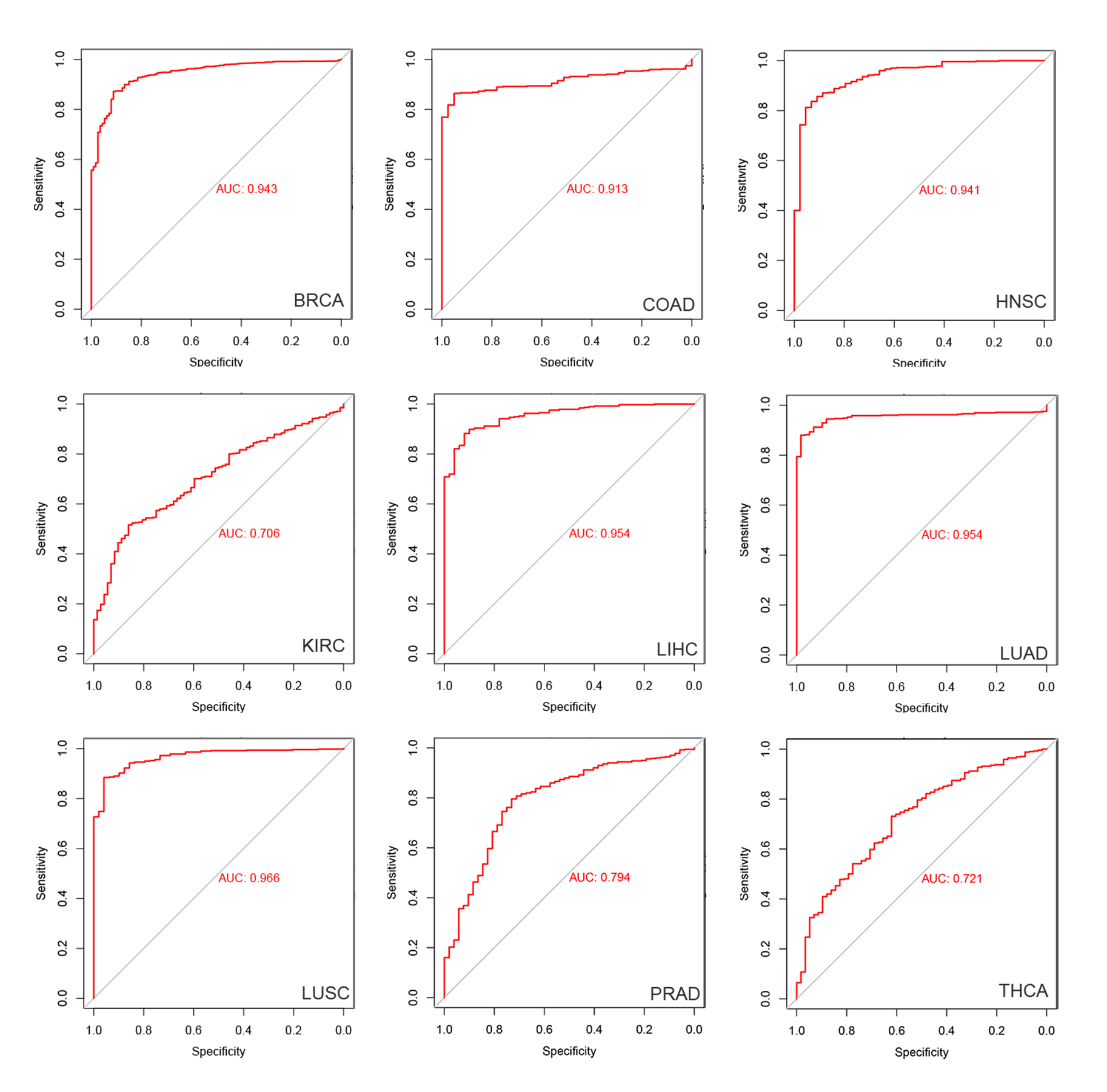

Supplement: Supplementary file 6 — Additional file 6: Fig. S3. The AUC of the ROC in BRCA, COAD, HNSC, KIRC, LIHC, LUAD, LUSC, PRAD, and THCA. [file 12935_2021_2432_MOESM6_ESM.tif]

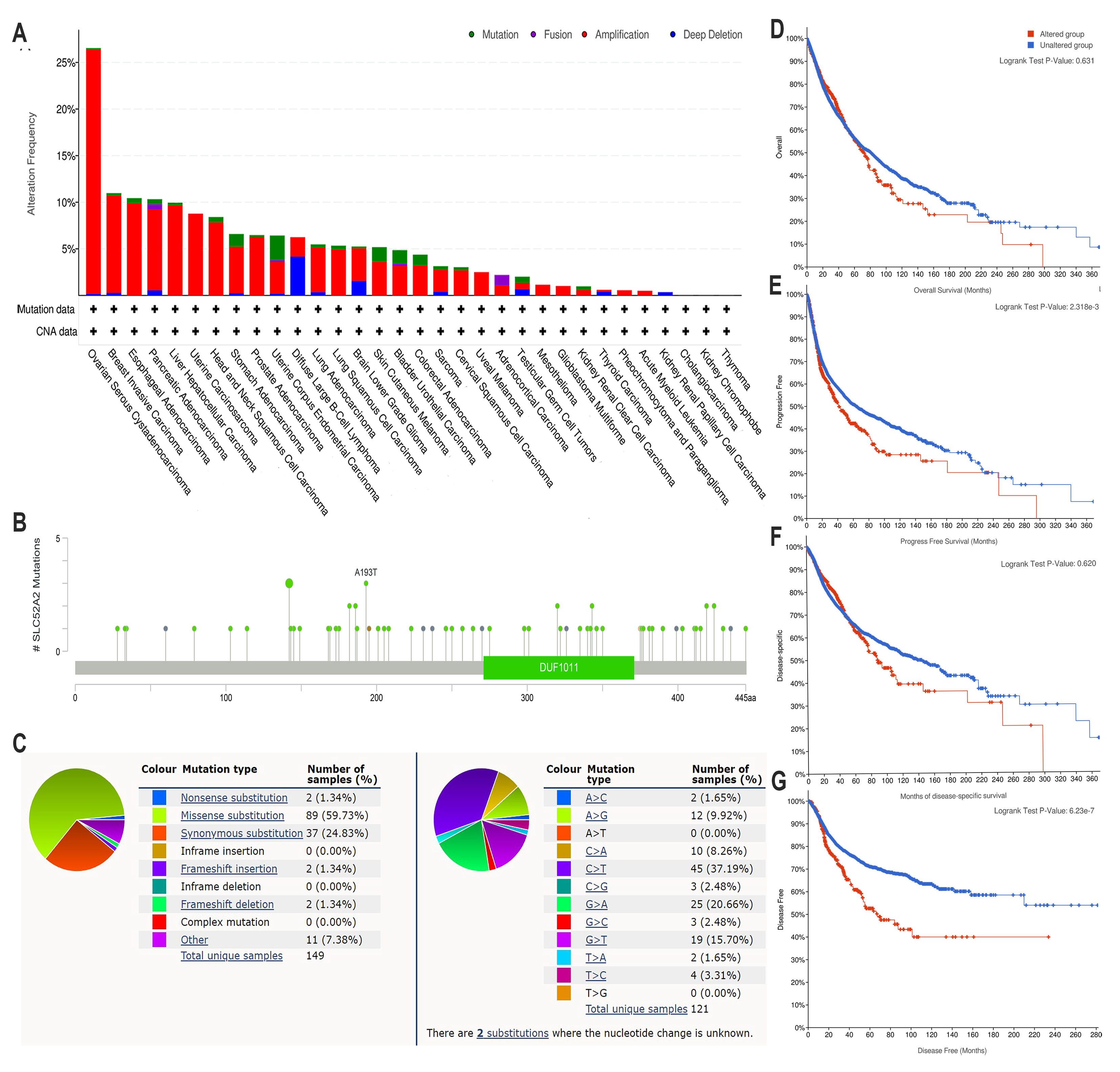

Supplement: Supplementary file 7 — Additional file 7: Fig. S4. SLC52A2 mutation landscape. (A) SLC52A mutation level from the cBioPortal database. (B) Mutation diagram of SLC52A2 in different cancer types across protein domains from the cBioPortal database. (C) Pie chart showing the percentage of the different mutation types of SLC52A2 in cancers according to the COSMIC database. (D-G) The genetic alteration of SLC52A2 and the survival prognosis of cancers by the cBioPortal database. [file 12935_2021_2432_MOESM7_ESM.tif]

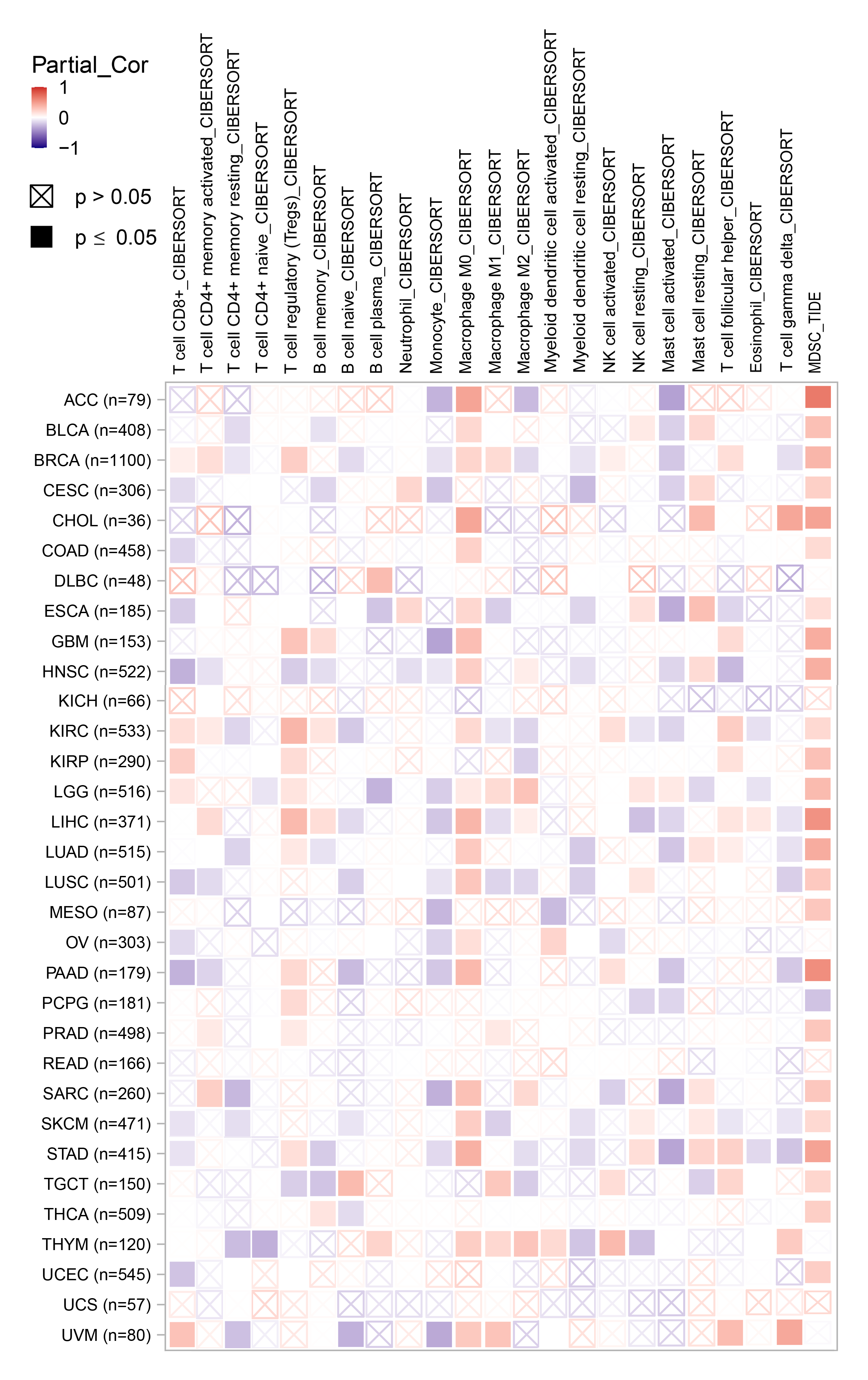

Supplement: Supplementary file 8 — Additional file 8: Fig. S5. the correlation between SLC52A2 expression and immune cell infiltration using CIBERSORT and TIDE algorithms. [file 12935_2021_2432_MOESM8_ESM.tif]

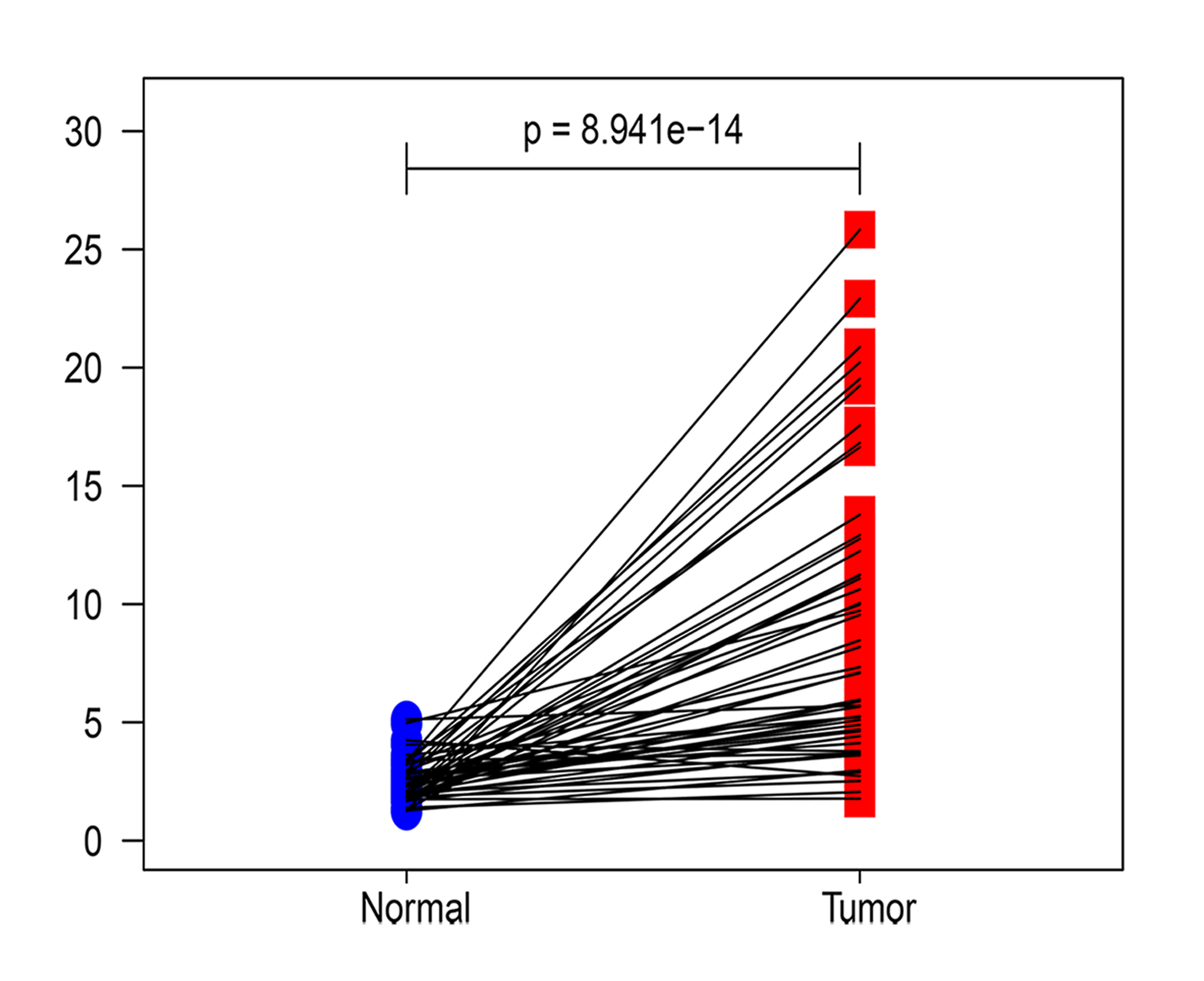

Supplement: Supplementary file 9 — Additional file 9: Fig. S6. Pairwise difference analysis of SLC52A2 expression in the TCGA-LIHC cohort. [file 12935_2021_2432_MOESM9_ESM.tif]

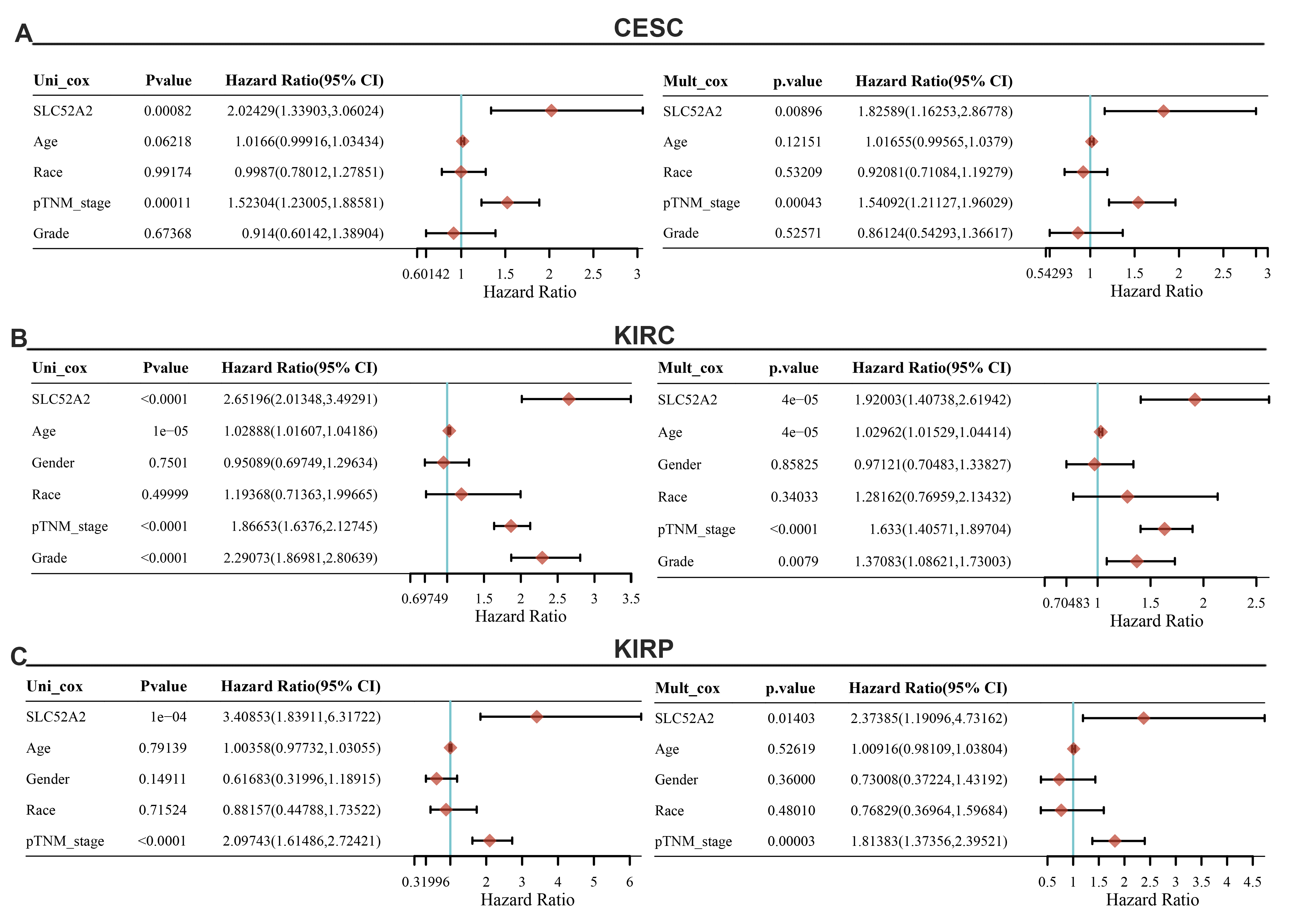

Supplement: Supplementary file 10 — Additional file 10: Fig. S7. Hazard ratio and P-value of constituents involved in univariate and multivariate Cox regression and some parameters of the SLC52A2 genes in CESC(A), KIRC (B), and KIRP(C). [age (years) is a continuous variable; Gender: Male vs Female (reference values); Race: Black/White vs Asian (reference values); pTNM stage: III/IV vs I/II (reference values); Grade: G3/G4 vs G1/G2 (reference values)]. [file 12935_2021_2432_MOESM10_ESM.tif]

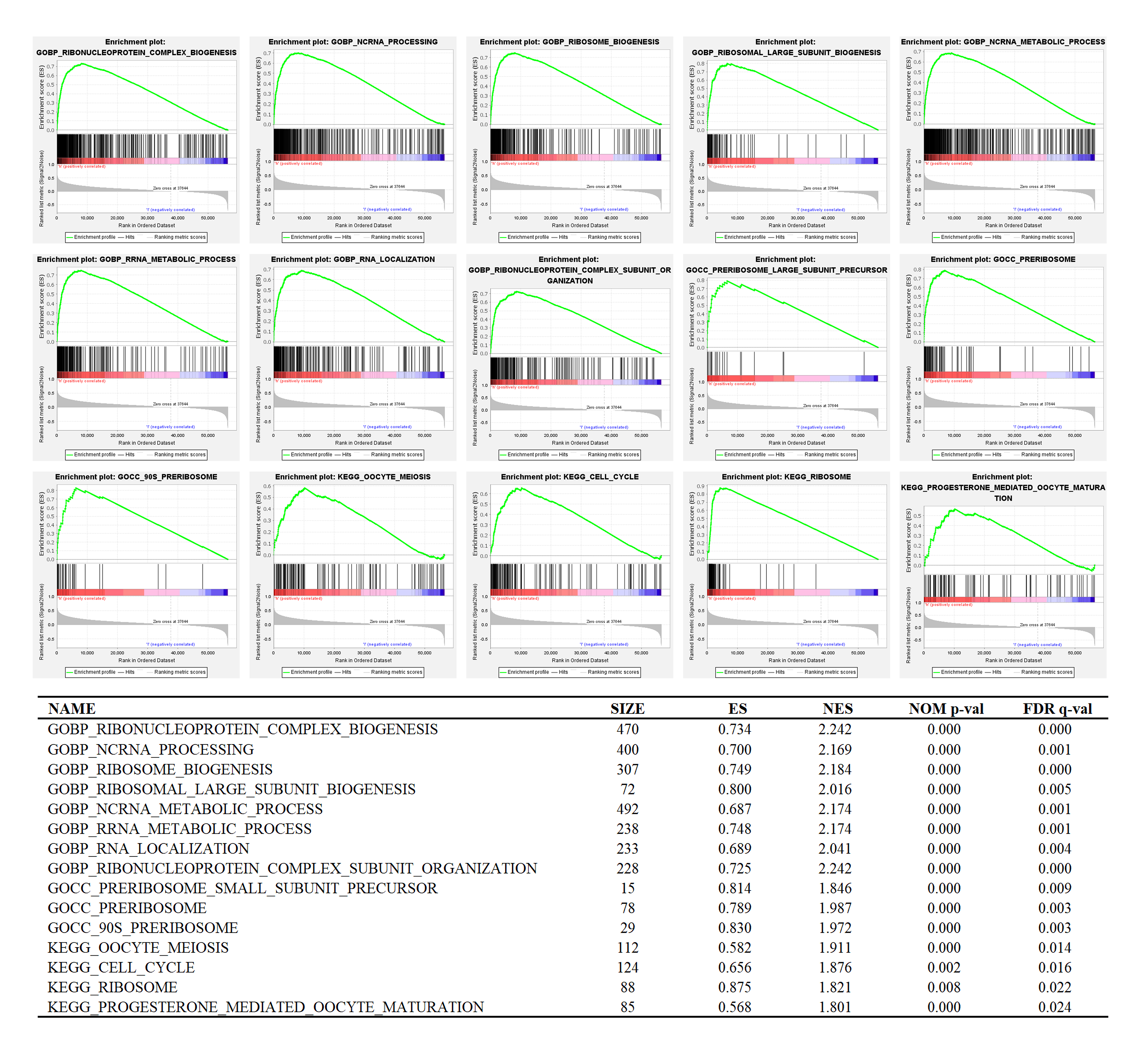

Supplement: Supplementary file 11 — Additional file 11: Fig. S8. KEGG and GEO enrichment analysis in the LIHC-TCGA cohort. KEGG, Kyoto Encyclopedia of Genes and Genomes; GO, Gene Ontology; BP, biological process; CC, cellular component. [file 12935_2021_2432_MOESM11_ESM.tif]

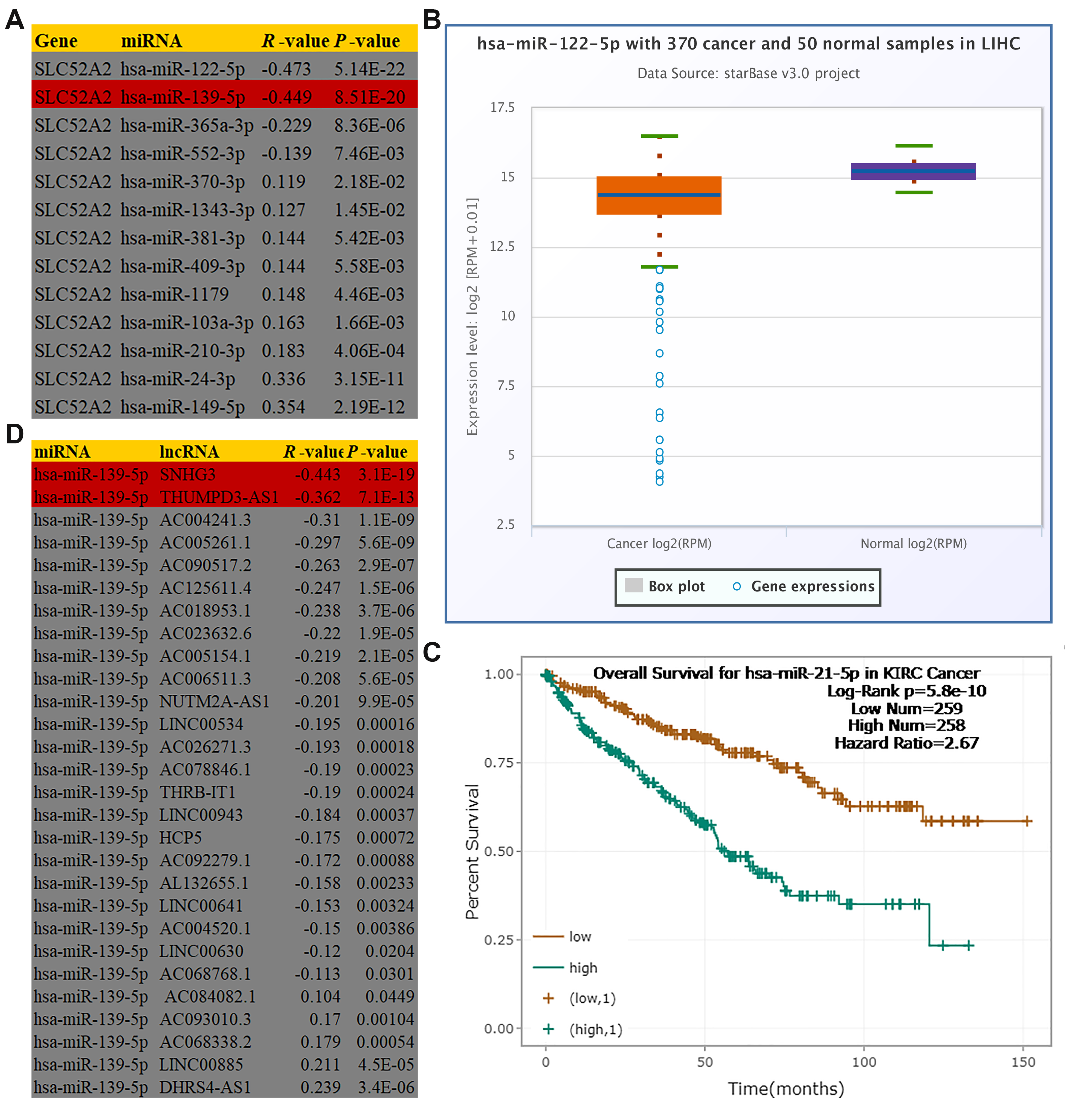

Supplement: Supplementary file 12 — Additional file 12: Fig. S9. Prediction and analysis of upstream lncRNA and miRNAs of SLC52A2 in Hepatocellular carcinoma. (A) Correlation analysis of SLC52A2 and miRNA using starbase database. (B) Analysis of hsa-miR-122-5p expression in hepatocellular carcinoma and paraneoplastic tissue using starbase database. (C) Correlation of hsa-miR-122-5p with prognosis using the starbase database. (D) Correlation analysis of hsa-miR-139-5p and lncRNA using starbase database. [file 12935_2021_2432_MOESM12_ESM.tif]
